# Dietary Diversity in the Eastern Mediterranean Region Before and During the COVID-19 Pandemic: Disparities, Challenges, and Mitigation Measures

**DOI:** 10.3389/fnut.2022.813154

**Published:** 2022-02-15

**Authors:** Maha Hoteit, Hussein Mortada, Ayoub Al-Jawaldeh, Rania Mansour, Batoul Yazbeck, Majid AlKhalaf, Khlood Bookari, Reema Tayyem, Narmeen J. Al-Awwad, Haleama Al Sabbah, Leila Cheikh Ismail, Radwan Qasrawi, Rania Abu Seir, Iman Kamel, Somaia Dashti, Sabika Allehdan, Mariam Al-Mannai, Hiba Bawadi, Mostafa Waly, The Regional CORONA COOKING Survey Group, Charlotte De Backer

**Affiliations:** ^1^Faculty of Public Health, Lebanese University, Beirut, Lebanon; ^2^PHENOL Research Group (Public HEalth Nutrition prOgram Lebanon), Faculty of Public Health, Lebanese University, Beirut, Lebanon; ^3^Lebanese University Nutrition Surveillance Center (LUNSC), Lebanese Food Drugs and Chemical Administrations, Lebanese University, Beirut, Lebanon; ^4^Faculty of Science IV, Lebanese University, Beirut, Lebanon; ^5^World Health Organization Regional Office for the Eastern Mediterranean, Cairo, Egypt; ^6^National Nutrition Committee, Saudi Food and Drug Authority, Riyadh, Saudi Arabia; ^7^Department of Clinical Nutrition, Faculty of Applied Medical Sciences, Taibah University, Madinah, Saudi Arabia; ^8^Department of Human Nutrition, College of Health Sciences, Qatar University, Doha, Qatar; ^9^Department of Clinical Nutrition and Dietetics, Faculty of Applied Health Sciences, The Hashemite University, Zarqa, Jordan; ^10^Department of Health Sciences, College of Natural and Health Sciences, Zayed University, Dubai, United Arab Emirates; ^11^Department of Clinical Nutrition and Dietetics, College of Health Sciences, University of Sharjah, Sharjah, United Arab Emirates; ^12^Nuffield Department of Women's & Reproductive Health, University of Oxford, Oxford, United Kingdom; ^13^Department of Computer Science, Al-Quds University, Jerusalem, Palestine; ^14^Department of Computer Engineering, Istinye University, Istanbul, Turkey; ^15^Faculty of Health Professions, Abu Dis, Palestine; ^16^National Research Centre, Cairo, Egypt; ^17^Public Authority for Applied Education and Training, Kuwait City, Kuwait; ^18^Department of Biology, College of Science, University of Bahrain, Zallaq, Bahrain; ^19^Department of Mathematics, College of Science, University of Bahrain, Zallaq, Bahrain; ^20^Human Nutrition Department, College of Health Sciences, QU-Health, Qatar University, Doha, Qatar; ^21^Food Science and Nutrition Department, College of Agricultural and Marine Sciences, Sultan Qaboos University, Muscat, Oman

**Keywords:** COVID-19 pandemic, dietary diversity, Eastern Mediterranean region, food consumption score, mitigation measures

## Abstract

The COVID-19 pandemic has revealed the Eastern Mediterranean Region's food system's fragility posing severe challenges to maintaining healthy sustainable lifestyle. The aim of this cross-sectional study (*N* = 13,527 household's family members, mean age: 30.3 ±11.6, 80% women) is to examine the impact of the COVID-19 pandemic on food consumption patterns and household's dietary diversity in 10 Eastern Mediterranean countries. A food frequency questionnaire was used to investigate the consumption patterns along with the calculation of the Food Consumption Score (FCS), a proxy indicator of dietary diversity. Data collected on cooking attitudes, shopping and food stock explore the community mitigation measures. In the overall population, before and during the pandemic, most food groups were consumed less or equal to 4 times per week. As evident from our findings and considering that the pandemic may be better, but it's not over, small to moderate changes in food consumption patterns in relatively short time periods can become permanent and lead to substantial poor dietary diversity over time. While it is a priority to mitigate the immediate impact, one area of great concern is the long-term effects of this pandemic on dietary patterns and dietary diversity in Eastern Mediterranean households. To conclude, the COVID-19 crisis revealed the region's unpreparedness to deal with a pandemic. While the aggressive containment strategy was essential for most countries to help prevent the spread, it came at a high nutritional cost, driving poor dietary diversity.

## Introduction

Prior to the unprecedented COVID-19 epidemic, nearly 690 million individuals worldwide consumed fewer calories than required (1). Beyond food deprivation, due to many reasons, a growing number of people have been forced to cut back the quantity and quality of the food they ingest (1). The physical and mental health repercussions of such deficit illustrate the indisputable public health importance of food consumption patterns, dietary diversity, and related food insecurity (1). During the COVID-19 pandemic, an estimation exceeding 280 million people were at risk of becoming food insecure (2). The loss of livelihoods due to COVID-19, caused food supply disruptions and income loss, limiting the access to nutritious food, and making households across the globe facing difficulties to have access to healthy diets (1). Consequently, more than 1.5 billion people couldn't afford a nutri-dense diet that meets the required essential nutrients and around 3 billion people faced difficulties in affording the cheapest healthy diet (1–3). At regional level, embedded with many challenges, the Eastern Mediterranean (EM) countries are faced with scarce and dwindling natural resources amidst high urbanization rates, populations increase, wars, climate changes, sociopolitical crises (4) and recently, the COVID-19 pandemic (5). Today, amid the COVID-19 pandemic, almost 54.5 million people are witnessing severe food insecurity in the region, along with an increase of four times in the percentage of hungry people in the Middle Eastern countries (6). Currently, in 12 countries including Algeria, Libya, Tunisia, Lebanon, Palestine, and Sudan, more than 10 million people were assisted by short-term assistances and cash-based transfers. In addition, the situation is extremely worsening and worrying in many countries affected by conflicts, violence and socioeconomic crises including Iraq, Libya, Somalia, Syria, Sudan, Yemen, and Lebanon (7, 8). Upon the exponential increase in COVID-19 consequences on the financial status of consumers, food insecurity started to aggravate in most EM countries. Households tend to change their food consumption patterns involuntarily, rely on savings, sell household durable assets and livestock, buy foods with high shelf-life, eat less, buy cheaper food, and limit food types they cannot afford such as meat and fish, and start consuming higher quantities of starchy food due to their wide availability and cheap prices (9). Households were forced to change their food consumption patterns as a mean of mitigation measures. The lack of studies concerning the changes in food consumption, dietary diversity, and mitigation measures had made this issue of a high priority. Thus, the aim of this study is to examine the impact of the COVID-19 pandemic on the food consumption patterns, the dietary diversity, and the mitigations measures among EM households residing in 10 Eastern Mediterranean countries.

## Materials and Methods

The online survey consisted of a cross-sectional study that was launched originally in 38 different countries. The Eastern Mediterranean regional data related to Lebanon and 9 other Arabic countries (Bahrain, Egypt, Jordan, Kuwait, Oman, Qatar, Saudi Arabia, United Arab Emirates, and Palestine) that have participated to this survey has been selected for the sake of analysis in this study. Questions of the survey were available in native Arabic language as well as other languages extending choices for the respondents. The survey was kept open between April 17th and June 25th, 2020 and consisted of multiple blocks of information. Participants included in this study were of age exceeding 18 years old, of both genders, residing in any of these Arabic countries. Convenience sampling was used to recruit respondents and advertisement of the survey was done using different social network platforms as well as academic networks of the research team. The questionnaire consisted of a validated online survey, that took around 20 min to be completed. A full overview of the study protocol, the questionnaire validation and the survey is accessible via https://osf.io/nz9xf/files/. It was used to collect information related to different topics including: sociodemographic and economic information, lockdown measures, mental health, cooking attitudes, shopping, food stock, and food frequency consumption in term of food portions per week (The question asked was: “how often did you eat the following (portions of) foods? Please indicate how often you consumed at least one portion of the following foods and drinks”). Regarding questions related to cooking attitudes, shopping, and food frequency consumption, respondents were asked to answer each question twice, reporting thus their behavior before the COVID-19 pandemic and during the COVID-19 lockdown. Food Consumption Score (FCS), which is a proxy indicator used for investigating the dietary diversity, was calculated using the frequency of consumption of different food groups consumed by a household during the 7 days before the survey. The calculation formula of the score FCS is: (starches × 2) + (pulses × 3) + vegetables+ fruit+ (meat × 4) + (dairy products × 4) + (fats × 0.5) + (sugar × 0.5). Prior to calculating the FCS score, response options were merged forming the following two categories: “lower than or equal to 4 times a week” and “equals five times a week or more” (10). The FCS was calculated for each of the respondents based on his answers to the food frequency questionnaire. We then multiplied the frequency by the weight of the food (as listed in the formula of FCS). Two different FCS were calculated, the first one was based on the answers of respondents about food frequency consumption before the lockdown and the second one was based on their answers during the lockdown. Everyone was then classified as having a high FCS (if it is >42) or low FCS (<42) (10). When interpreting these results, people who tend to have higher FCS (>42) were consuming a diversified diet, this achieving a diversified balanced diet (10).

### Statistical Analysis

Respondents' characteristics were presented as frequencies (percentages) for categorical variables while means ± standard deviation (SD) were used for continuous variables. Results were assessed for all participants as well as for countries separately in order to detect any potential behavior difference between them. Different statistical tests were used: Chi-square test was used to investigate differences for categorical variables between groups while independent *t*-test was applied for continuous variables and Marginal Homogeneity test was used to differentiate between paired data (comparison before and during COVID-19). To look for factors that may impact the FCS, a binary logistic regression was calculated. In this regression, the FCS (high vs. low) was the dependent variable. A backward approach was used and factors having a *p*-value <0.05 were considered as significant. Odds Ratio (OR) and its confidence interval were also calculated for each of the factors. A *p*-value lower than 0.05 was considered significant. Statistical analysis was conducted on IBM SPSS Statistics for Mac, Version 24.

### Ethical Consideration

A consent form was attached at the beginning of the online survey that protects participants, let them know their rights and responsibilities and keep their information confidential. The study was conducted 126 according to the guidelines of the Declaration of Helsinki, and approved by the Ethical Committee for the Social Sciences and Humanities of the University of Antwerp (file number 20_46) as well as in all other concerned countries. The patients/participants provided their written informed consent to participate in this study.

## Results

### Socio-Demographic and Economic Characteristics

The total number of respondents who filled completely the survey in the 10 Arab countries was 13,527 households' family members. They were thus used for the subsequent analysis. Among them, 80% were females. Most participants were either adults (24 to 64 years old, 56.7%) or youth (19 to 24 years old, 37.6%). However, a very low percentage of adolescents (18 years) (4.7%) and elder people (1%) had registered (Table 1). The mean age of the respondents was 30.3 years with a SD of 11.69. Males who responded to this survey were significantly older than females (*p*-value = 0) (Table 1). Regarding the distribution of the respondents among the different countries, the higher percentage was recorded in Saudi Arabia (22.8%) while the lowest percentage was for Oman (1.4%). However, it is to be noted that a similar percentage of respondents has been recorded for the MENA region (Middle East and North Africa countries including Lebanon, Jordan, Egypt, and Palestine; 48.4%) and for the Gulf Cooperation Council (GCC) countries (the remaining 6 countries; 51.6%). As regards to the educational level, around 59% of the respondents had a bachelor's degree when analyzing both genders together. In addition, a significant higher percentage (60.8%) of females had bachelor's degree compared with males (51.9%) (*p*-value = 0) (Table 1). The household composition was also analyzed before and during the lockdown in which most households were composed of three to five adults (more than 40%). This trend has been also observed when considering males and females each separately (Table 1). When looking to economic characteristics, a similar percentage of respondents were students (39.2%) and active workers (39.7%) while the minority were unemployed (21.1%) before the COVID-19 lockdown. The percentage of unemployed individuals had increased to 33.4% during the lockdown. This increase has been also observed for males (7.8% before lockdown to 20.9% during lockdown) and females (24.4% before lockdown to 36.4% during lockdown). Moreover, the COVID-19 lockdown has also induced a loss of income among 39.4% of respondents. This loss was significantly higher (*p*-value = 0) for men (47.3%) compared to women (37.5%). In addition, most respondents, when taken either all together (58.4%) or categorized as males (57.9%) or females (58.6%) each alone, struggle to make money last until the end of the month or to earn enough money for shopping (52.4, 49.5, and 53.3% for all respondents, men and women respectively) (Table 1).

**Table 1:** sociodemographic and socioeconomic characteristics of the studied population.

**Variables**	**Overall *N* (%)**	**Male *N* (%)**	**Female *N* (%)**	***p*** **-value**
Age (mean ± SD)	30.30 ±11.69	33.13 ± 13.15	29.61 ±11.20	
Adolescents (18 years)	18	18	18	
Youth	21.02 ± 1.31	20.94 ± 1.32	21.03 ± 1.31	0
Adult	36.81 ± 10.09	37.97 ± 10.74	36.47 ± 9.86	0
Elderly	68.58 ± 5.09	68.19 ± 4.90	68.92 ± 5.26	
**Age categories (%)**				
Adolescents (18 years)	638 (4.7)	135 (5.1)	503 (4.6)	
Youth	5,087 (37.6)	709 (26.7)	4,378 (40.3)	0
Adult	7,667(56.7)	1,749 (65.9)	5,918 (54.4)	
Elderly	135 (1)	63 (2.3)	72 (0.7)	
**Gender (%)**				
Male	2,656 (19.6)	NA	NA	
Female	10,871 (80.4)			
**Countries (%)**				
Bahrain	693 (5.1)	126 (4.7)	567 (5.2)	0.001
Egypt	734 (5.4)	170 (6.4)	564 (5.2)	
Jordan	2,675 (19.8)	581 (21.9)	2,094 (19.3)	
Kuwait	728 (5.4)	156 (5.9)	572 (5.2)	
Lebanon	2,282 (16.9)	436 (16.4)	1846 (17.0)	
Oman	186 (1.4)	32 (1.2)	154 (1.4)	
Qatar	653 (4.8)	135 (5.1)	518 (4.8)	
Saudi Arabia	2,999 (22.2)	530 (19.9)	2,469 (22.7)	
United Arab Emirates	1,718 (12.7)	313 (11.8)	1,405 (12.9)	
Palestine	859 (6.3)	177 (6.7)	682 (6.3)	
**Education (%)**				
Under a high school diploma	698 (5.1)	166 (6.2)	532 (4.9)	0
High school diploma or equivalent	2,763 (20.4)	553 (20.8)	2,210 (20.3)	
Bachelor's degree	7,991 (59.1)	1,378 (51.9)	6,613 (60.8)	
Master's degree	1,537 (11.4)	358 (13.5)	1,179 (10.9)	
Doctorate	538 (4.0)	201 (7.6)	337 (3.1)	
**Number of adults living in the same household before the lockdown (%)**				
<3	1,649 (38.0)	340 (39.7)	1,309 (37.6)	0.393
3–5	1,860 (42.9)	364 (42.5)	1,496 (43.0)	
More than 5	829 (19.1)	152 (17.8)	677 (19.4)	
**Number of adults living in the same household during the lockdown (%)**				
<3	4,851 (36.5)	1,114 (42.6)	3,737 (35.0)	0
3–5	5,993 (45.0)	1,096 (41.9)	4,897 (45.8)	
More than 5	2,459 (18.5)	405 (15.5)	2,054 (19.2)	
Employment before the lockdown (%)				Overall: 0
Student	5,296 (39.2)	775 (29.2)	4,521 (41.6)	Students: 0
Working	5,375 (39.7)	1,673 (63.0)	3,702 (34.0)	Working: 0
Didn't working	2,856 (21.1)	208 (7.8)	2,648 (24.4)	Not working: 0
Employment during the lockdown (%)				Overall: 0
Student	4,805 (35.5)	676 (25.4)	4,129 (38.0)	Students: 0
Working	4,210 (31.1)	1,425 (53.7)	2,785 (25.6)	Working: 0
Didn't working	4,512 (33.4)	555 (20.9)	3,957 (36.4)	Not working: 0
**Loss of income since lockdown (%)**				
Yes	5,336 (39.4)	1,256 (47.3)	4,080 (37.5)	0
No	8,191 (60.6)	1,400 (52.7)	6,791 (62.5)	
**Struggle to make money last until the end of month (%)**				
No	5,621 (41.6)	1,119 (42.1)	4,502 (41.4)	0.501
Yes	7,906 (58.4)	1,537 (57.9)	6,369 (58.6)	
**Struggle to have enough money to shopping (%)**				
No	6,420 (47.5)	1,340 (50.5)	5,080 (46.7)	0.001
Yes	7,107 (52.5)	1,316 (49.5)	5,791 (53.3)	

### Consumption of Food Groups and Food Consumption Score

Tables 2–5 show the food groups consumption and the FCS in the overall population, by region and by country.

**Table 2:** Food groups consumption among countries in the GCC and MENA regions, before and during COVID-19 pandemic.

		**Overall** ***N*** **(%)**			**GCC** ***N*** **(%)**			**MENA** ***N*** **(%)**			**Comparison MENA vs. GCC**	
**Food groups consumed**	**Frequency per week**	**Before**	**During**	***p*** **-value**	**Before**	**During**	***p*** **-value**	**Before**	**During**	***p*** **-value**	***p*** **-value**	
											**Before COVID-19**	**During-COVID-19**
Fruit (fresh or frozen)	4 or less	8,499 (62.8)	8,410 (62.2)	0.093	4,689 (67.2)	4,429 (63.5)	0	3,810 (58.2)	3,981 (60.8)	0	0	0.001
	5 or more	5,028 (37.2)	5,117 (37.8)		2,288 (32.8)	2,548 (36.5)		2,740 (41.8)	2,569 (39.2)			
Vegetables (fresh or frozen)	4 or less	6,162 (45.6)	6,704 (49.6)	0	3,475 (49.8)	3,570 (51.2)	0.017	2,687 (41)	3,134 (47.8)	0	0	0
	5 or more	7,365 (54.4)	6,823 (50.4)		3,502 (50.2)	3,407 (48.8)		3,863 (59)	3,416 (52.2)			
Legumes/pulses (e.g., beans, lentils, chickpeas)	4 or less	11,248 (83.2)	10,863 (80.3)	0	5,807 (83.2)	5,615 (80.5)	0	5,441 (83.1)	5,248 (80.1)	0	0.801	0.602
	5 or more	2,279 (16.8)	2,664 (19.7)		1,170 (16.8)	1,362 (19.5)		1,109 (16.9)	1,302 (19.9)			
Nuts	4 or less	10,829 (80.1)	10,704 (79.1)	0.009	5,441 (78)	5,338 (76.5)	0.003	5,388 (82.3)	5,366 (81.9)	0.51	0	0
	5 or more	2,698 (19.9)	2,823 (20.9)		1,536 (22)	1,639 (23.5)		1,162 (17.7)	1,184 (18.1)			
Processed meat/poultry/fish/ vegetarian alternatives	4 or less	10,358 (76.6)	11,226 (83)	0	5,146 (73.8)	5,599 (80.2)	0	5,212 (79.6)	5,627 (85.9)	0	0	0
	5 or more	3,169 (23.4)	2,301 (17)		1,831 (26.2)	1,378 (19.8)		1338 (20.4)	923 (14.1)			
Unprocessed fish	4 or less	12,319 (91.1)	12,003 (88.7)	0	6,230 (89.3)	6,030 (86.4)	0	6,089 (93)	5,973 (91.2)	0	0	0
	5 or more	1208 (8.9)	1524 (11.3)		747 (10.7)	947 (13.6)		461 (7)	577 (8.8)			
Unprocessed poultry	4 or less	10,292 (76.1)	10,165 (75.1)	0.009	4,974 (71.3)	4,893 (70.1)	0.027	5,318 (81.2)	5,272 (80.5)	0.163	0	0
	5 or more	3,235 (23.9)	3,362 (24.9)		2,003 (28.7)	2,084 (29.9)		1,232 (18.8)	1,278 (19.5)			
Unprocessed red meat^*^	4 or less	11,663 (86.2)	11,339 (83.8)	0	5,907 (84.7)	5,671 (81.3)	0	5,756 (87.9)	5,668 (86.5)	0	0	0
	5 or more	1,864 (13.8)	2,188 (16.2)		1,070 (15.3)	1,306 (18.7)		794 (12.1)	882 (13.5)			
Whole wheat bread, pasta, grains	4 or less	9,878 (73)	9,486 (70.1)	0	4,848 (69.5)	4,699 (67.3)	0	5,030 (76.8)	4,787 (73.1)	0	0	0
	5 or more	3,649 (27)	4,041 (29.9)		2,129 (30.5)	2,278 (32.7)		1,520 (23.2)	1,763 (26.9)			
White bread, pasta, grains	4 or less	8,837 (65.3)	8,535 (63.1)	0	4,541 (65.1)	4,361 (62.5)	0	4,296 (65.6)	4,174 (63.7)	0.001	0.539	0.142
	5 or more	4,690 (34.7)	4,992 (36.9)		2,436 (34.9)	2,616 (37.5)		2,254 (34.4)	2,376 (36.3)			
Milk	4 or less	8,402 (62.1)	8,304 (61.4)	0.039	3,891 (55.8)	3,884 (55.7)	0.865	4,511 (68.9)	4,420 (67.5)	0.004	0	0
	5 or more	5,125 (37.9)	5,223 (38.6)		3,086 (44.2)	3,093 (44.3)		2,039 (31.1)	2,130 (32.5)			
Other dairy products	4 or less	6,421 (47.5)	6,550 (48.4)	0.014	3,292 (47.2)	3,286 (47.1)	0.893	3,129 (47.8)	3,264 (49.8)	0	0.494	0.001
	5 or more	7,106 (52.5)	6,977 (51.6)		3,685 (52.8)	3,691 (52.9)		3,421 (52.2)	3,286 (50.2)			
Sweet snacks	4 or less	9,440 (69.8)	9,176 (67.8)	0	4,616 (66.2)	4,466 (64)	0	4,824 (73.6)	4,710 (71.9)	0.002	0	0
	5 or more	4,087 (30.2)	4,351 (32.2)		2,361 (33.8)	2,511 (36)		1,726 (26.4)	1,840 (28.1)			
Sugared beverages	4 or less	7,962 (58.9)	8,062 (59.6)	0.051	4,343 (62.2)	4,321 (61.9)	0.564	3,619 (55.3)	3,741 (57.1)	0.001	0	0
	5 or more	5,565 (41.1)	5,465 (40.4)		2,634 (37.8)	2,656 (38.1)		2,931 (44.7)	2,809 (42.9)			
Fats and oils	4 or less	10,499 (77.6)	10,190 (75.3)	0	5,299 (75.9)	5,073 (72.7)	0	5,200 (79.4)	5,117 (78.1)	0.014	0	0
	5 or more	3,028 (22.4)	3,337 (24.7)		1,678 (24.1)	1,904 (27.3)		1,350 (20.6)	1,433 (21.9)			

* *Unprocessed meats: (refers to all mammalian muscle meat including beef, veal, pork, lamb, mutton, horse, and goat)*.

**Table 3:** Food groups consumption among countries in the GCC region, before and during COVID-19 pandemic.

		**Bahrain N (%)**			**Kuwait N (%)**			**Oman N (%)**			**Qatar N (%)**			**Saudi Arabia N (%)**			**United Arab Emirates N (%)**		
		**Before**	**During**	***p*** **-value**	**Before**	**During**	***p*** **-value**	**Before**	**During**	***p*** **-value**	**Before**	**During**	***p*** **-value**	**Before**	**During**	***p*** **-value**	**Before**	**During**	***p*** **-value**
Fruit (fresh or frozen)	4 or less	403 (58.2)	380 (54.8)	0.071	454 (62.4)	470 (64.6)	0.211	81 (43.5)	73 (39.2)	0.216	388 (59.4)	366 (56)	0.086	2,240 (74.7)	2,140 (71.4)	0	1,123 (65.4)	1,000 (58.2)	0
	5 or more	290 (41.8)	313 (45.2)		274 (37.6%)	258 (35.4)		105 (56.5)	113 (60.8)		265 (40.6)	287 (44)		759 (25.3)	859 (28.6)		595 (34.6)	718 (41.8)	
Vegetables (fresh or frozen)	4 or less	308 (44.4)	312 (45)	0.8	316 (43.4)	364 (50)	0	54 (29)	57 (30.6)	0.735	274 (42)	295 (45.2)	0.085	1,660 (55.4)	1,703 (56.8)	0.102	863 (50.2)	839 (48.8)	0.265
	5 or more	385 (55.6)	381 (55)		412 (56.6)	364 (50)		132 (71)	129 (69.4)		379 (58)	358 (54.8)		1,339 (44.6)	1,296 (43.2)		855 (49.8)	879 (51.2)	
Legumes/ pulses	4 or less	595 (85.9)	583 (84.1)	0.281	603 (82.8)	573 (78.7)	0.012	152 (81.7)	151 (81.2)	1	526 (80.6)	524 (80.2)	0.92	2,554 (85.2)	2,483 (82.8)	0.001	1,377 (80.2)	1,301 (75.7)	0
	5 or more	98 (14.1)	110 (15.9)		125 (17.2)	155 (21.3)		34 (18.3)	35 (18.8)		127 (19.4)	129 (19.8)		445 (14.8)	516 (17.2)		341 (19.8)	417 (24.3)	
Nuts	4 or less	493 (71.1)	475 (68.5)	0.142	520 (71.4)	535 (73.5)	0.238	130 (69.9)	141 (75.8)	0.054	509 (77.9)	492 (75.3)	0.149	2,460 (82)	2,410 (80.4)	0.025	1,329 (77.4)	1,285 (74.8)	0.014
	5 or more	200 (28.9)	218 (31.5)		208 (28.6)	193 (26.5)		56 (30.1)	45 (24.2)		144 (22.1)	161 (24.7)		539 (18)	589 (19.6)		389 (22.6)	433 (25.2)	
Processed meat/poultry/ fish/ vegetarian alternatives	4 or less	523 (75.5)	559 (80.7)	0.005	528 (72.5)	560 (76.9)	0.015	139 (74.7)	162 (87.1)	0.001	490 (75)	529 (81)	0.001	2,206 (73.6)	2,440 (81.4)	0	1,260 (73.3)	1,349 (78.5)	0
	5 or more	170 (24.5)	134 (19.3)		200 (27.5)	168 (23.1)		47 (25.3)	24 (12.9)		163 (25)	124 (19)		793 (26.4)	559 (18.6)		458 (26.7)	369 (21.5)	
Unprocessed fish	4 or less	598 (86.3)	570 (82.3)	0.006	659 (90.5)	646 (88.7)	0.171	152 (81.7)	153 (82.3)	1	598 (91.6)	569 (87.1)	0	2,737 (91.3)	2,655 (88.5)	0	1,486 (86.5)	1,437 (83.6)	0.002
	5 or more	95 (13.7)	123 (17.7)		69 (9.5)	82 (11.3)		34 (18.3)	33 (17.7)		55 (8.4)	84 (12.9)		262 (8.7)	344 (11.5)		232 (13.5)	281 (16.4)	
Unprocessed poultry	4 or less	502 (72.4)	477 (68.8)	0.03	565 (77.6)	551 (75.7)	0.239	146 (78.5)	138 (74.2)	0.23	484 (74.1)	479 (73.4)	0.707	1,960 (65.4)	1,961 (65.4)	1	1,317 (76.7)	1,287 (74.9)	0.111
	5 or more	191 (27.6)	216 (31.2)		163 (22.4)	177 (24.3)		40 (21.5)	48 (25.8)		169 (25.9)	174 (26.6)		1,039 (34.6)	1,038 (34.6)		401 (23.3)	431 (25.1)	
Unprocessed red meat	4 or less	605 (87.3)	597 (86.1)	0.396	622 (85.4)	606 (83.2)	0.089	163 (87.6)	159 (85.5)	0.556	555 (85)	535 (81.9)	0.027	2,500 (83.4)	2,367 (78.9)	0	1,462 (85.1)	1,407 (81.9)	0.001
	5 or more	88 (12.7)	96 (13.9)		106 (14.6)	122 (16.8)		23 (12.4)	27 (14.5)		98 (15)	118 (18.1)		499 (16.6)	632 (21.1)		256 (14.9)	311 (18.1)	
Wholewheat bread, pasta, grains	4 or less	499 (72)	476 (68.7)	0.087	519 (71.3)	497 (68.3)	0.105	123 (66.1)	127 (68.3)	0.671	468 (71.7)	452 (69.2)	0.205	2,082 (69.4)	2,046 (68.2)	0.18	1,157 (67.3)	1,101 (64.1)	0.008
	5 or more	194 (28)	217 (31.3)		209 (28.7)	231 (31.7)		63 (33.9)	59 (31.7)		185 (28.3)	201 (30.8)		917 (30.6)	953 (31.8)		561 (32.7)	617 (35.9)	
White bread, pasta, grains	4 or less	475 (68.5)	436 (62.9)	0.002	508 (69.8)	476 (65.4)	0.015	114 (61.3)	115 (61.8)	1	424 (64.9)	419 (64.2)	0.736	1,899 (63.3)	1,850 (61.7)	0.06	1,121 (65.3)	1,065 (62)	0.006
	5 or more	218 (31.5)	257 (37.1)		220 (30.2)	252 (34.6)		72 (38.7)	71 (38.2)		229 (35.1)	234 (35.8)		1,100 (36.7)	1,149 (38.3)		597 (34.7)	653 (38)	
Milk	4 or less	364 (52.5)	354 (51.1)	0.403	432 (59.3)	415 (57)	0.146	90 (48.4)	95 (51.1)	0.441	339 (51.9)	341 (52.2)	0.928	1,720 (57.4)	1,724 (57.5)	0.897	946 (55.1)	955 (55.6)	0.654
	5 or more	329 (47.5)	339 (48.9)		296 (40.7)	313 (43)		96 (51.6)	91 (48.9)		314 (48.1)	312 (47.8)		1,279 (42.6)	1,275 (42.5)		772 (44.9)	763 (44.4)	
Other dairy products	4 or less	326 (47)	323 (46.6)	0.867	318 (43.7)	326 (44.8)	0.565	77 (41.4)	73 (39.2)	0.607	295 (45.2)	303 (46.4)	0.536	1,413 (47.1)	1,427 (47.6)	0.587	863 (50.2)	834 (48.5)	0.145
	5 or more	367 (53)	370 (53.4)		410 (56.3)	402 (55.2)		109 (58.6)	113 (60.8)		358 (54.8)	350 (53.6)		1,586 (52.9)	1,572 (52.4)		855 (49.8)	884 (51.5)	
Sweet snacks	4 or less	496 (71.6)	487 (70.3)	0.481	430 (59.1)	413 (56.7)	0.184	145 (78)	136 (73.1)	0.2	433 (66.3)	443 (67.8)	0.444	1,978 (66)	1,886 (62.9)	0	1,134 (66)	1,101 (64.1)	0.112
	5 or more	197 (28.4)	206 (29.7)		298 (40.9)	315 (43.3)		41 (22)	50 (26.9)		220 (33.7)	210 (32.2)		1,021 (34)	1,113 (37.1)		584 (34)	617 (35.9)	
Sugared beverages	4 or less	435 (62.8)	423 (61)	0.315	427 (58.7)	421 (57.8)	0.642	121 (65.1)	122 (65.6)	1	393 (60.2)	386 (59.1)	0.585	1,810 (60.4)	1,850 (61.7)	0.098	1,157 (67.3)	1,119 (65.1)	0.056
	5 or more	258 (37.2)	270 (39)		301 (41.3)	307 (42.2)		65 (34.9)	64 (34.4)		260 (39.8)	267 (40.9)		1,189 (39.6)	1,149 (38.3)		561 (32.7)	599 (34.9)	
Fats and oils	4 or less	509 (73.4)	482 (69.6)	0.026	512 (70.3)	502 (69.0)	0.444	152 (81.7)	148 (79.6)	0.596	493 (75.5)	469 (71.8)	0.040	2,376 (79.2)	2,287 (76.3)	0	1,257 (73.2)	1,185 (69.0)	0
	5 or more	184 (26.6)	211 (30.4)		216 (29.7)	226 (31.0)		34 (18.3)	38 (20.4)		160 (24.5)	184 (28.2)		623 (20.8)	712 (23.7)		461 (26.8)	533 (31.0)	

**Table 4:** Food groups consumption among countries in the MENA region, before and during COVID-19 pandemic.

		**Egypt** ***N (%)***			**Jordan** ***N (%)***			**Lebanon** ***N (%)***			**Palestine** ***N (%)***		
		**Before**	**During**	***p*-value**	**Before**	**During**	***p*-value**	**Before**	**During**	***p*-value**	**Before**	**During**	***p*-value**
Fruit (fresh or frozen)	4 or less	346 (47.1)	324 (44.1)	0.086	1,821 (68.1)	1,836 (68.6)	0.538	1,178 (51.6)	1,300 (57)	0	465 (54.1)	521 (60.7)	0
	5 or more	388 (52.9)	410 (55.9)		854 (31.9)	839 (31.4)		1104 (48.4)	982 (43)		394 (45.9)	338 (39.3)	
Vegetables (fresh or frozen)	4 or less	290 (39.5)	283 (38.6)	0.625	1,233 (46.1)	1,448 (54.1)	0	822 (36)	979 (42.9)	0	342 (39.8)	424 (49.4)	0
	5 or more	444 (60.5)	451 (61.4)		1,442 (53.9)	1,227 (45.9)		1,460 (64)	1,303 (57.1)		517 (60.2)	435 (50.6)	
Legumes/pulses	4 or less	614 (83.7)	584 (79.6)	0.008	2,257 (84.4)	2,195 (82.1)	0.004	1,848 (81)	1,775 (77.8)	0.001	722 (84.1)	694 (80.8)	0.027
	5 or more	120 (16.3)	150 (20.4)		418 (15.6)	480 (17.9)		434 (19)	507 (22.2)		137 (15.9)	165 (19.2)	
Nuts	4 or less	609 (83)	577 (78.6)	0.003	2,173 (81.2)	2,147 (80.3)	0.239	1,920 (84.1)	1,947 (85.3)	0.147	686 (79.9)	695 (80.9)	0.497
	5 or more	125 (17)	157 (21.4)		502 (18.8)	528 (19.7)		362 (15.9)	335 (14.7)		173 (20.1)	164 (19.1)	
Processed meat/poultry/ fish/vegetarian alternatives	4 or less	617 (84.1)	650 (88.6)	0.003	2,044 (76.4)	2,262 (84.6)	0	1,857 (81.4)	1,991 (87.2)	0	694 (80.8)	724 (84.3)	0.022
	5 or more	117 (15.9)	84 (11.4)		631 (23.6)	413 (15.4)		425 (18.6)	291 (12.8)		165 (19.2)	135 (15.7)	
Unprocessed fish	4 or less	688 (93.7)	676 (92.1)	0.134	2,441 (91.3)	2,376 (88.8)	0	2,152 (94.3)	2,140 (93.8)	0.366	808 (94.1)	781 (90.9)	0.003
	5 or more	46 (6.3)	58 (7.9)		234 (8.7)	299 (11.2)		130 (5.7)	142 (6.2)		51 (5.9)	78 (9.1)	
Unprocessed poultry	4 or less	613 (83.5)	593 (80.8)	0.06	2,010 (75.1)	1,989 (74.4)	0.387	2,032 (89)	2,033 (89.1)	1	663 (77.2)	657 (76.5)	0.683
	5 or more	121 (16.5)	141 (19.2)		665 (24.9)	686 (25.6)		250 (11)	249 (10.9)		196 (22.8)	202 (23.5)	
Unprocessed red meat	4 or less	643 (87.6)	624 (85)	0.048	2,307 (86.2)	2,255 (84.3)	0.009	2,043 (89.5)	2,041 (89.4)	0.948	763 (88.8)	748 (87.1)	0.18
	5 or more	91 (12.4)	110 (15)		368 (13.8)	420 (15.7)		239 (10.5)	241 (10.6)		96 (11.2)	111 (12.9)	
Sweet snacks	4 or less	583 (79.4)	552 (75.2)	0.007	1,931 (72.2)	1,829 (68.4)	0	1,664 (72.9)	1,734 (76)	0.001	646 (75.2)	595 (69.3)	0
	5 or more	151 (20.6)	182 (24.8)		744 (27.8)	846 (31.6)		618 (27.1)	548 (24)		213 (24.8)	264 (30.7)	
Wholewheat bread, pasta, grains	4 or less	535 (72.9)	519 (70.7)	0.195	2,092 (78.2)	1,966 (73.5)	0	1,757 (77)	1,694 (74.2)	0.003	646 (75.2)	608 (70.8)	0.008
	5 or more	199 (27.1)	215 (29.3)		583 (21.8)	709 (26.5)		525 (23)	588 (25.8)		213 (24.8)	251 (29.2)	
White bread, pasta, grains	4 or less	482 (65.7)	433 (59)	0	1,767 (66.1)	1,728 (64.6)	0.122	1,491 (65.3)	1,484 (65)	0.79	556 (64.7)	529 (61.6)	0.063
	5 or more	252 (34.3)	301 (41)		908 (33.9)	947 (35.4)		791 (34.7)	798 (35)		303 (35.3)	330 (38.4)	
Milk	4 or less	318 (43.3)	298 (40.6)	0.045	1,962 (73.3)	1,892 (70.7)	0.001	1,678 (73.5)	1,693 (74.2)	0.433	553 (64.4)	537 (62.5)	0.164
	5 or more	416 (56.7)	436 (59.4)		713 (26.7)	783 (29.3)		604 (26.5)	589 (25.8)		306 (35.6)	322 (37.5)	
Other dairy products	4 or less	277 (37.7)	277 (37.7)	1	1,399 (52.3)	1,416 (52.9)	0.488	1,039 (45.5)	1,155 (50.6)	0	414 (48.2)	416 (48.4)	0.941
	5 or more	457 (62.3)	457 (62.3)		1,276 (47.7)	1,259 (47.1)		1,243 (54.5)	1,127 (49.4)		445 (51.8)	443 (51.6)	
Sugared beverages	4 or less	422 (57.5)	428 (58.3)	0.634	1,494 (55.9)	1,516 (56.7)	0.371	1,282 (56.2)	1,359 (59.6)	0	421 (49)	438 (51)	0.216
	5 or more	312 (42.5)	306 (41.7)		1,181 (44.1)	1,159 (43.3)		1,000 (43.8)	923 (40.4)		438 (51)	421 (49)	
Fats and oils	4 or less	637 (86.8)	612 (83.4)	0.017	2,081 (77.8)	1,979 (74.0)	0	1,814 (79.5)	1,878 (82.3)	0.001	668 (77.8)	648 (75.4)	0.123
	5 or more	97 (13.2)	122 (16.6)		594 (22.2)	696 (26.0)		468 (20.5)	404 (17.7)		191 (22.2)	211 (24.6)	

**Table 5:** Food consumption score of studied population before and during the COVID-19 pandemic.

**Main Outcome**		**Bahrain *N* (%)**	**Egypt *N* (%)**	**Jordan *N* (%)**	**Kuwait *N* (%)**	**Lebanon *N* (%)**	**Oman *N* (%)**	**Qatar *N* (%)**	**KSA *N* (%)**	**UAE *N* (%)**	**Palestine *N* (%)**	***p*** **-value**	**EMR *N* (%)**	**GULF *N* (%)**	**MENA *N* (%)**	***p*** **-value**
FCS before COVID-19		107.7 ± 43.4	104.8 ± 44.3	93.8 ± 46.3	105.9 ± 46.6	93.9 ± 41.9	117.5 ± 41.9	108.1 ± 44	101.6 ± 47.3	106 ± 48	96.8 ± 46.1	0	100.3 ± 45.9	104.8 ± 46.7	95.5 ± 44.7	0
FCS during COVID-19		111.2 ± 48	109.5 ± 44.7	93.6 ± 50.8	105.3 ± 51.7	88.1 ± 44.4	112.1 ± 41.7	107.9 ± 48.6	101.7 ± 51.4	108 ± 51.2	96.5 ± 50.3	0	99.8 ± 49.8	105.5 ± 50.7	93.9 ± 48.3	0
*p*-value		0.003	0	0.783	0.616	0	0.011	0.869	0.791	0.013	0.838		0.138	0.086	0	
FCS-before COVID-19	Low	38 (5.5)	62 (8.4)	333 (12.4)	48 (6.6)	211 (9.2)	8 (4.3)	40 (6.1)	287 (9.6)	139 (8.1)	90 (10.5)	0	1,256 (9.3)	560 (8.0)	696 (10.6)	0
	High	655 (94.5)	672 (91.6)	2,342 (87.6)	680 (93.4)	2,071 (90.8)	178 (95.7)	613 (93.9)	2,712 (90.4)	1,579 (91.9)	769 (89.5)		12,271 (90.7)	6,417 (92)	5,854 (89.4)	
FCS-during COVID-19	Low	38 (5.5)	48 (6.5)	411 (15.4)	68 (9.3)	315 (13.8)	10 (5.4)	45 (6.9)	340 (11.3)	147 (8.6)	118 (13.7)	0	1,540 (11.4)	648 (9.3)	892 (13.6)	0
	High	655 (94.5)	686 (93.5)	2,264 (84.6)	660 (90.7)	1,967 (86.2)	176 (94.6)	608 (93.1)	2,659 (88.7)	1,571 (91.4)	741 (86.3)		11,987 (88.6)	6,329 (90.7)	5,658 (86.4)	
Percentage of decline in the NA FCS		0	−1.90	3	2.7	4.6	1.10	0.8	1.70	0.50	3.2		2.1	1.30	3	
*p*-value		1	0.099	0	0.011	0	0.625	0.472	0.001	0.516	0.004		0	0	0	

#### Fruits Group

In the overall population, fruits consumption (fresh or frozen), did not differ during the lockdown compared to the period before (*p*-value = 0.09). Despite that more than 60% of the EMR population consume fruits lower than or equal to 4 times a week, it was observed that the percentage of people consuming fruits equals five times a week or more was relatively higher in the MENA region (41.8% before the lockdown and 39.2% during the lockdown) compared to the GCC countries (32.8% before the pandemic and 36.5% during the lockdown) before and during the lockdown (*p*-value = 0.000 and *p*-value = 0.001, respectively). In addition, the percentage of people consuming fruits equals five times a week or more increased during the lockdown of 4% in the GCC countries (*p*-value = 0.000) and decreased of 2.6% in the MENA region (*p*-value = 0.000) compared to the period preceding the pandemic (Table 2).

When analyzed by country, it appeared that before and during the lockdown, the lowest consumption of fruits was observed in Kuwait, Saudi Arabia, UAE, and Jordan (Tables 3, 4). In another word, the percentage of people consuming fruits equals five times a week or more was low in these countries compared to Bahrain (41.8% before the lockdown and 45.2% during the lockdown), Oman (56.5% before the lockdown and 60.8% during the lockdown), Qatar (40.6% before and 44% during the lockdown), Egypt (52.9% before and 55.9% during the lockdown), Lebanon (48.4% before and 43% during the lockdown), and Palestine (45.9% before and 39.3% during the lockdown) (*p*-value = 0.000) (Tables 3, 4).

#### Vegetables Group

According to Table 2, it appears that half the population consumed vegetables equals five times a week or more before and during the pandemic. Like the fruits group consumption, it was observed that the percentage of people consuming vegetables frequently (equals five times a week or more), was higher in the MENA region before and during the lockdown (59 and 52.2%) compared to the GCC countries (50.2 and 48.8%) (*p*-value = 0.000). Furthermore, a decrease in vegetable's consumption of 4, 2, and 7% was observed during the pandemic in the overall population, the GCC, and the MENA countries, respectively (*p*-value = 0.000, *p*-value = 0.017, and *p*-value = 0.000, respectively). The analysis of data per country showed that the lowest intake was observed in Saudi Arabia (44.6% before the lockdown and 43.2% during the lockdown) and the highest intake was in Oman (71% before the lockdown and 69.4% during the lockdown) (*p*-value = 0.000). Furthermore, during the pandemic, there was a decrease of vegetables intake of 6% in Kuwait (*p*-value = 0.000), of 9% in Jordan (*p*-value = 0.000), of 7% in Lebanon (*p*-value = 0.000), and of 10% in Palestine (*p*-value = 0.000). However, it remained unchanged in other countries (Tables 3, 4).

#### Legumes and Pulses Group

More than 80% of people living in the EM countries, in the current study, where consuming legumes and pulses lower than or equal to 4 times a week. Nevertheless, the percentage of people consuming frequently legumes and pulses increased during the pandemic of 3% in the overall population, the GCC and the MENA region (*p*-value = 0.000, *p*-value = 0.000 and *p*-value = 0.000, respectively). When comparing the consumption of legumes and pulses between GCC and MENA countries, before and during the lockdown, no difference was observed (*p*-value = 0.8 and *p*-value = 0.6, respectively) (Table 2). Furthermore, the intake of legumes and pulses, before and during the pandemic, were around 20% or less in all the countries studied. An increase ranging between 2 and 4% of legumes and pulses intake was observed in Saudi Arabia (*p*-value = 0.001), Jordan (*p*-value = 0.004), Lebanon (*p*-value = 0.001), Palestine (*p*-value = 0.027), Kuwait (*p*-value = 0.012), UAE (*p*-value = 0.000), and Egypt (*p*-value = 0.008) (Tables 3, 4).

#### Nuts Group

Three quarters of the population were consuming nuts lower than or equal to 4 times a week. As per Table 2, the percentage of people consuming frequently nuts and derivatives was higher in the GCC countries (22% before the lockdown and 23.5% during the lockdown) compared to the MENA countries (17.7% before the lockdown and 18.1% during the lockdown) before and during the lockdown (*p*-value = 0.000 and *p*-value = 0.000, respectively). A slight significant increase (around 1%) in the consumption of nuts group was observed during the pandemic in the overall population and in the GCC countries only (*p*-value = 0.000 and *p*-value = 0.000, respectively). This was not the case in the MENA countries (*p*-value = 0.51). Per country, a slight increase of 1.6% was observed in Saudi Arabia (*p*-value = 0.025), of 3% in UAE (*p*-value = 0.014) and of 4% in Egypt (*p*-value = 0.003) only. The lowest consumption of nuts group was observed in Lebanon (15.9% before the lockdown and 14.7% during the lockdown) and the highest intake was in Bahrain (29% before the lockdown and 31.5% during the lockdown) (Tables 3, 4).

#### Processed Meat/Poultry/Fish/Vegetarian Alternatives

A range between 14 and 26% of people living in the EM countries were consuming frequently (equals five times a week or more) processed meat/poultry/fish and vegetarian alternatives, before and during the pandemic. The consumption of this food group was higher in the GCC countries before and during the pandemic (26.2 and 19.8%, respectively) compared to the MENA countries (20.4% and 14.1%, respectively) (*p*-value = 0.000 and *p*-value = 0.000). A decrease of 6% in the consumption of this food group was observed during the lockdown in the EM countries together and in each GCC and MENA countries alone (*p*-value = 0.000, *p*-value = 0.000 and *p*-value = 0.000, respectively) (Table 2). The per-country analysis of data on processed meat/poultry/fish/vegetarians' alternatives intake showed a decrease of 5% in Bahrain (*p*-value = 0.005), 4% in Kuwait (*p*-value = 0.015), 12% in Oman (*p*-value = 0.001), 6% in Qatar (*p*-value = 0.001), 8% in Saudi Arabia (*p*-value = 0.000), 5% in UAE (*p*-value = 0.000), 4% in Egypt (*p*-value = 0.003), 8% in Jordan (*p*-value = 0.000), 6% in Lebanon (*p*-value = 0.000), and 4% in Palestine (*p*-value = 0.022) (Tables 3, 4).

#### Unprocessed Fish, Unprocessed Poultry, and Unprocessed Meats

It was observed that <30% of the population ate frequently unprocessed fish, poultry, and meats. The frequency of consumption of this food group was higher in the GCC countries compared to the MENA countries, before and during the pandemic (*p*-value = 0.000 and *p*-value = 0.000, respectively). A slight significant increase (1–3%) was observed in the frequency of consumption of this group during the pandemic in the EM countries together (fish-*p*-value = 0.000, poultry-*p*-value = 0.009 and meat-*p*-value = 0.000) and in the GCC countries (fish-*p*-value = 0.000, poultry-*p*-value-0.027 and meat-*p*-value = 0.000). Same trend was observed in the MENA countries, except for the consumption of poultry group which remained unchanged before and during the pandemic (*p*-value = 0.16) (Table 2). The lowest consumption of unprocessed fish (5.7% before and 6.2% during the lockdown), poultry (11% before and 10.9% during the lockdown), and meats (10.5% before and 10.6% during the lockdown) was observed in Lebanon (Table 4). The intake of fish increased during the pandemic in a range between 2 and 4% significantly in Bahrain (*p*-value = 0.006), in Qatar (*p*-value = 0.000), in Saudi Arabia (*p*-value = 0.00), in UAE (*p*-value = 0.002), in Jordan (*p*-value = 0.000), and in Palestine (*p*-value = 0.003) and remained unchanged in the other countries (Tables 3, 4). The intake of poultry had increased around 4% during the pandemic in Bahrain only (*p*-value = 0.03) and remained stable in all the remaining countries. The percentage of people consuming red meats equals five times a week or more, increased during the pandemic in Egypt (3%, *p*-value = 0.048), Jordan (5%, *p*-value = 0.009), Qatar (3%, *p*-value = 0.027), Saudi Arabia (4%, *p*-value = 0.000), and UAE (3%, *p*-value = 0.001) only.

#### White and Whole Wheat, Bread, Pasta, and Grains

Only 30% of the EM population in this study were consuming wholewheat bread, pasta, and grains in a frequency equals five times a week or more. An increase of 3% was observed during the pandemic (*p*-value = 0.00). The frequency of consumption of this food group in the GCC countries (30.5% before the lockdown and 32.7% during the lockdown) was higher than that observed in the MENA region (23.2% before the lockdown and 26.9% during the lockdown) (*p*-value = 0.000 and *p*-value = 0.000, respectively). On the other hand, the percentage of people consuming white bread, pasta, and grains were relatively high in the MENA countries as well as in GCC countries compared to those who consumed whole grains frequently. Furthermore, no significant differences were observed between the frequent consumption of these two food groups among the GCC and the MENA countries (*p*-value = 0.5 and *p*-value = 0.1, respectively) (Table 2). Before the pandemic, the highest intake of wholewheat and white breads/pasta and grains was observed in Oman (33.9% before and 38.7% during the lockdown, respectively) and during the lockdown the highest intake of this group was observed in Palestine (38.4%) and in Egypt (41%) (*p*-value = 0.000). It was observed that the consumption of wholewheat food groups increased during the lockdown of 3% in UAE (*p*-value = 0.008), 5% in Jordan (*p*-value = 0.00), 2% in Lebanon (*p*-value = 0.003), and 5% in Palestine during the lockdown (*p*-value = 0.008) and remained unchanged in the other countries. However, the consumption of white breads/pasta and grains increased during the lockdown of 6% in Bahrain (*p*-value = 0.002), of 4% in Kuwait (*p*-value = 0.015) of 2% in Saudi Arabia (*p*-value = 0.06) and of 7% in Egypt (*p*-value = 0.000) (Tables 3, 4).

#### Milk and Dairy Products Group

More than half the population were consuming milk and dairy products lower than or equal to 4 times a week. The consumption of milk and dairy products, during the pandemic, were higher in the GCC countries (44.3 and 52.9%) compared to the MENA countries (32.5 and 50.2%) (*p*-value = 0.000 and *p*-value =0.001, respectively). Overall, during the lockdown, a slight increase was remarkable in the consumption of milk (0.6%, *p*-value = 0.03) along with a slight decrease in the consumption of other dairy products e.g., Cheese and yogurt (0.9%, *p*-value = 0.014) (Table 2). The analyses per country showed an increase in the milk intake of 3% in Egypt (*p*-value = 0.045) and Jordan (*p*-value = 0.001) and a decrease in other dairy products consumption of 5% in Lebanon (*p*-value = 0.000) (Tables 3, 4).

#### Sugar Group (Products and Beverages)

A range between 40 and 45% of the population studied consumed this group equals five times a week or more. Despite the increase in the frequency of consumption of sugary beverages products in the studied EM countries (*p*-value = 0.000), in the GCC countries (*p*-value = 0.000) and in the MENA countries (*p*-value = 0.002), more than 36% of the population consumed this group 4 times and more, per week (Table 2). An increase of 3–6% in the consumption of sweet products was observed during the lockdown in Saudi Arabia (*p*-value = 0.000), Egypt (*p*-value = 0.007), Jordan (*p*-value = 0.000), and Palestine (*p*-value = 0.000) along with a decrease of 3% in Lebanon (*p*-value = 0.001). The intake of sugary beverages did not differ between the 2 periods of time except in Lebanon in which the consumption decreased of 3% (*p*-value = 0.000) (Tables 3, 4).

#### Fats and Oils Group

It was observed that added fats and oils were less frequently consumed in the EM countries studied (around 25% of the total population). A slight increase of 1–4% was observed among the EMR, GCC, and MENA countries (*p*-value = 0.000, *p*-value = 0.000 and *p*-value = 0.014, respectively). All in all, the consumption of added fats and oils was higher in the GCC compared to the MENA countries, before (24 vs. 20%; *p*-value = 0.000) and during the lockdown (27 vs. 22%; *p*-value = 0.000) (Table 2). At the GCC countries level, the consumption of food groups increased of 3% during the lockdown in Bahrain (*p*-value = 0.026), in Qatar (*p*-value = 0.04), in Saudi Arabia (*p*-value = 0.00), and in UAE (*p*-value = 0.00). Similarly, an increase of up to 4% was observed in Egypt (*p*-value = 0.017) and Jordan (*p*-value = 0.00) along with a decrease of 3% in Lebanon (*p*-value = 0.001). Prior to and during the lockdown, the lowest intake of added fats and oils was observed in Egypt (13 vs. 16%, respectively) and the highest was in Kuwait (around 30% in both study periods) (Tables 3, 4).

**Figure 1:**
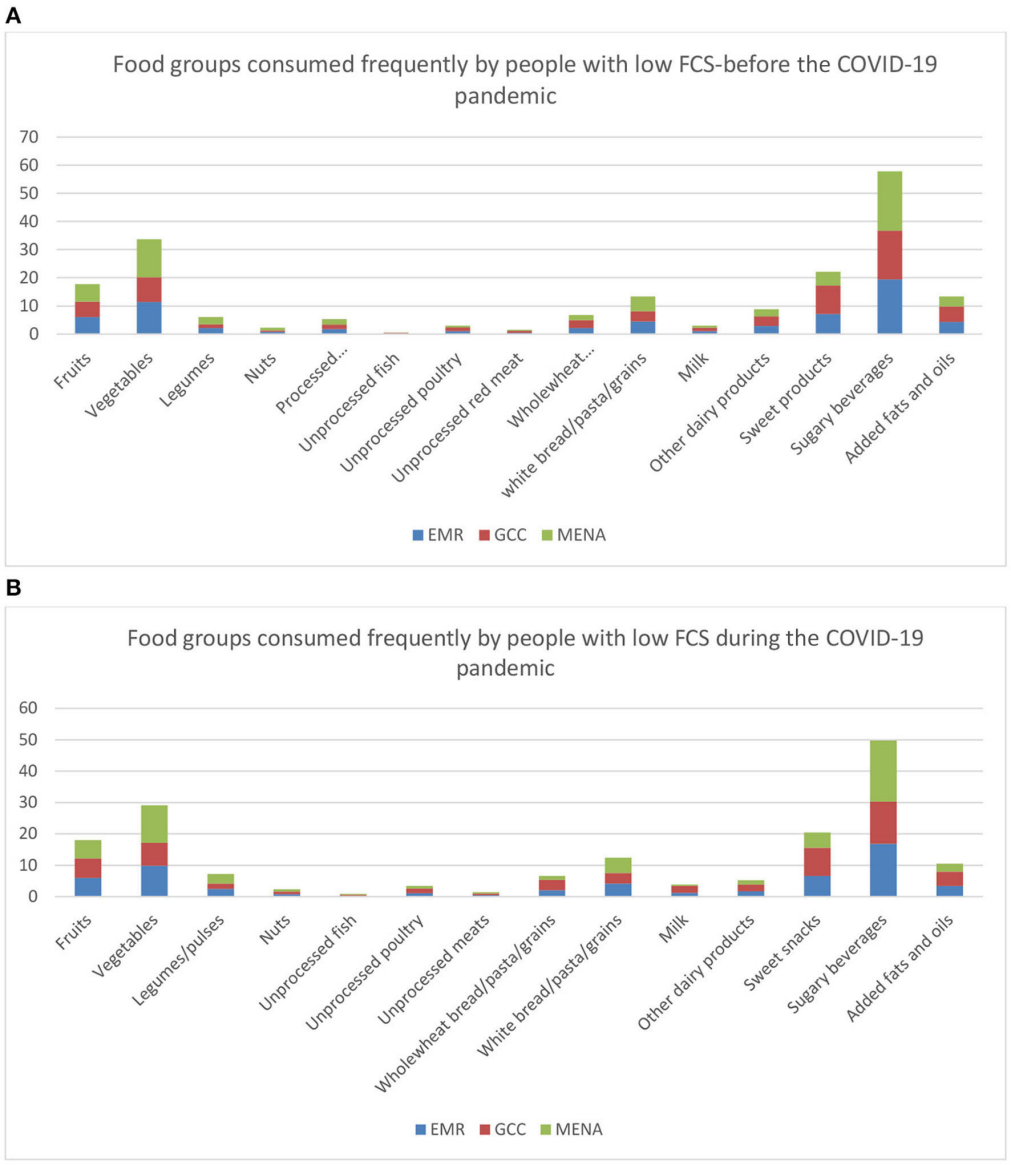
**(A)** Food groups consumed frequently by people having low FCS in the EM countries prior to the pandemic. **(B)** Food groups consumed frequently by people having low FCS in the EM countries during the pandemic.

The Food Consumption Score was calculated based on the equation explained previously. Compared to the period preceding the pandemic, the mean levels, and SD of the FCS in all the countries studied was equal to 100.3 ± 45.9 and it remained unchanged during the pandemic (*p*-value = 0.13). Before the pandemic, the FCS in the GCC countries (104.8 ± 46.7) was higher than that of the MENA countries (95.5 ± 44.7) (*p*-value = 0.000). However, during the pandemic, the FCS declined in the MENA countries from 95.5 to 93.9 (*p*-value = 0.00) but remained unchanged in the GCC countries (*p*-value = 0.08). The lowest FCS values were observed in the MENA countries: Jordan, Lebanon, and Palestine (Table 5). The percentage of people having low FCS (<42) increased from 9.3 to 11.4% in the overall EM countries studied during the lockdown. Moreover, in the GCC and the MENA countries, the percentage of people having low FCS increased from to 8% to 9.3% and from 10.8 to 13.6%, respectively (*p*-value = 0.00 and *p*-value = 0.00, respectively). The percentage of people with high FCS decreased of 3% in Jordan, 2.7% in Kuwait, 4.6% in Lebanon, 1.7% in Saudi Arabia, and 3.2% in Palestine (*p*-value = 0.00) (Table 5). However, this was not significant for three countries which includes UAE, Oman, and Qatar. The percentage of people having high FCS increased insignificantly of 1.9% in Egypt (*p*-value = 0.09) and remained stable in Bahrain. Figures 1A,B showed the percentage of food groups consumed frequently by people with low FCS. These households were mainly dependent on sugary beverages and vegetables intake rather than other nutritious food groups. Same trends were observed before and during the pandemic (Figures 1A,B).

### Food Consumption Patterns and Mitigation Measures

#### Cooking Practices and Barriers

Table 3 shows that most cooking practices (11 out of 13) showed a significant increase during the lockdown. The highest increase was recorded for cooking with leftover foods (60.4% positive answers before lockdown compared to 65.7% during lockdown) (*p*-value = 0.00). The only practices that showed a significant decrease during lockdown was throwing away food leftovers (33.8% before lockdown compared to 32.5% during lockdown) (*p*-value = 0.00). It was observed that the attitudes of “planning a nutritious-varied diet” and “thinking about healthy choices” increased of 4.7% and 2.6% during the lockdown (*p*-value = 0.00 and *p*-value = 0.00), respectively, despite the null change in the practice of “cooking meals using healthy ingredients” (*p*-value = 0.3) and despite the null change also in “feeling confident about managing money to buy healthy food” (*p*-value = 0.6). Similarly, a significant increase of around 4.5% was observed in the practice of “reading of nutrition panels to make healthy choices” (*p*-value = 0.00). More than 65% of people were “changing recipes to make them healthier,” were “cooking with leftovers” and were avoiding “throwing away foods.” As for barriers against healthy cooking, almost 34–38% of people “didn't have the funds or the access for the foods/ingredients they needed or wanted to buy” and 28% of them “didn't have access to cooking facilities (stove, oven…)” (*p*-value = 0.00). At the level of the GCC and the MENA countries, we noticed a range between 23–47% and 20–50%, respectively, of people who were ignoring planning varied diet, avoiding thinking about healthy choices, disregard managing money to buy healthy food, pay no attention to cook meals at home using healthy ingredients, avoid changing recipes to make them healthier and ignore the nutrition panel to make healthy choices. Moreover, three quarters of the people living in GCC countries and 60% of those living in the MENA countries, were having monetary access to buy health food, were having access to food and also to cooking facilities. This trend decreases significantly in a range between 2 and 6% in the GCC countries and a range of 2% to 10% in the MENA countries, during the lockdown, ameliorating by this the cooking practices during this period of time in both regions (Supplementary Table S1). The analysis by gender shows the same significant trends as in the overall population (Data not shown). All in all, it was observed that during the pandemic, men witnessed a significant modification in cooking practices of which planning to consume healthy varied diet (*p*-value = 0.00), cooking healthy meals and recipes (*p*-value = 0.00) from leftovers (*p*-value = 0.00). Similar trend was also shown in women (Data not shown).

#### Criteria for Recipe Selection

With regards to the criteria for recipes selection, the lockdown due to the COVID-19 pandemic was found to significantly increase the percentage of agreeing responses for all criterions. For instance, during the lockdown, more than 70% of people selected their recipes with few ingredients (71.5%) that are easily available at home (79.4%) or at store (79.3%), inexpensive (68%), and healthy (72.8%) (Supplementary Table S2). Before the pandemic, more than half to three quarters the people living in the EM countries studied, were selecting recipes that were achievable with few ingredients, were available at home or can be easily found at the store, inexpensive to prepare, healthy, and cheap. During the lockdown, an increase ranging between 2 and 20%, was observed in these patterns (Supplementary Table S2).

#### Dietary Shopping Practices

Shopping practices have also been affected during the lockdown. Indeed, more than 60% of respondents agreed that they search more for cheapest prices before and during the lockdown. While, this increase was not very important and not significant, we observed a significant similar trend when analyzing by gender (*p*-value = 0.00) (Supplementary Table S3).

During the lockdown, respondents admitted significantly a reduction of 12.8% less going physically to select and buy food and that they preferred to order their food products online (variation of 1.2%) and have it delivered at home rather than being delivered at a seller's point (4.4%) (*p*-value = 0.00). Regarding places of groceries shopping, there was a significant decrease during the lockdown whatever was the place. In addition, it is remarkable that respondents had shown a disinterest of buying food at organic/ fair trade shops or specialty stores during lockdown. Before the pandemic, in the GCC countries, there was a decrease in shopping patterns of which a decrease of 17% in shopping foods physically from markets (*p*-value = 0.00), of 10% in shopping groceries from supermarkets (*p*-value = 0.001), of 6% in shopping from corner stores (*p*-value = 0.00), a range of 7% to 16% in shopping from farmer or organic stores, of 19% in shopping from specialties store (butcher, bakery, etc.…) (*p*-value = 0.00) and of 5% in buying meal boxes (*p*-value = 0.00). However, a 4% increase in making online food orders (*p*-value = 0.00) was observed. At the MENA countries, we noticed the same trends in shopping patterns, however, people living in these countries were making less orders online to buy groceries and reach markets more frequently compared to people living in the MENA countries (Supplementary Table S3).

#### Food Stock

**Figure 2:**
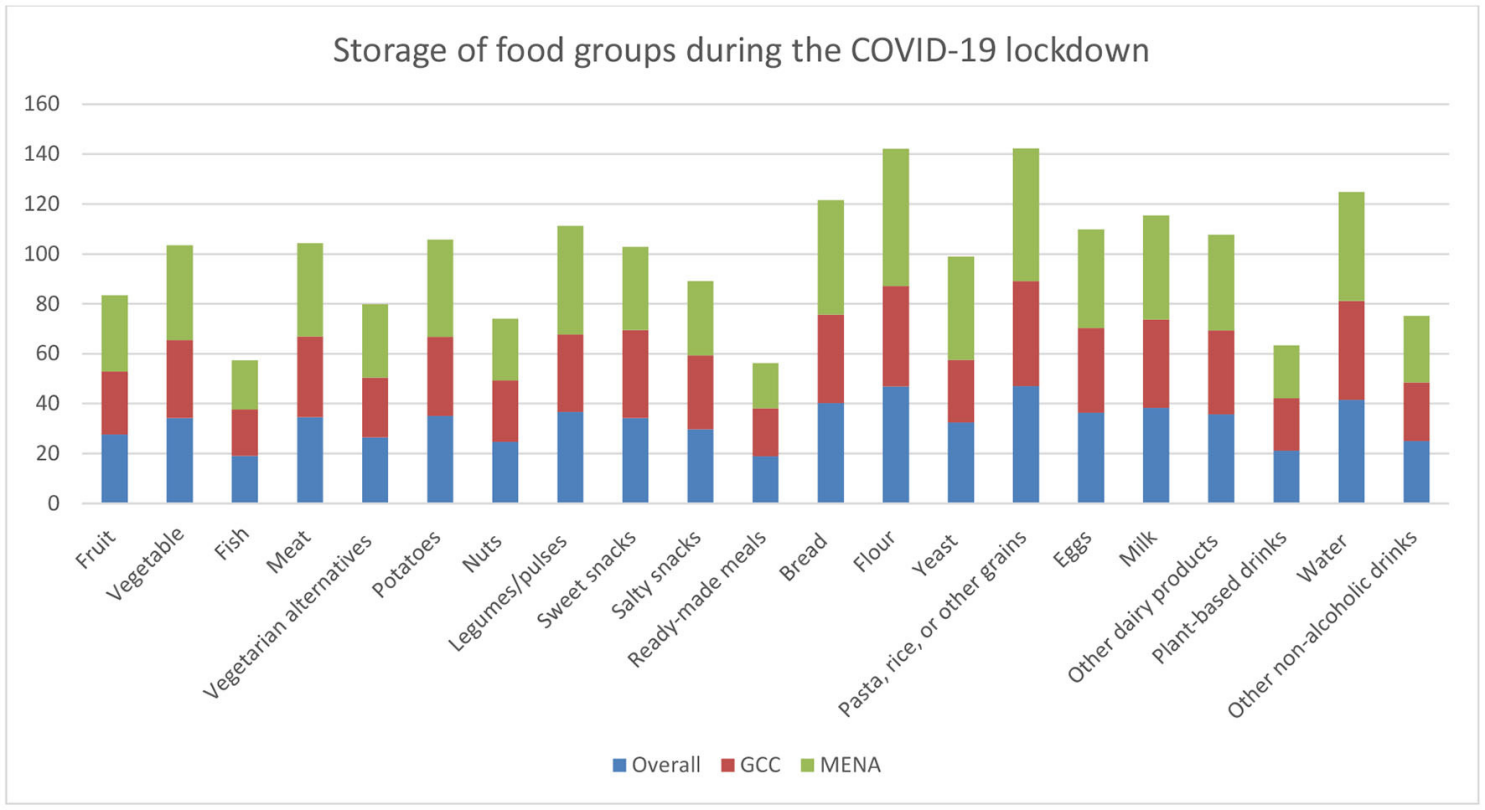
Food stock patterns during the pandemic, overall and by regions.

Food storage was also affected during the pandemic. Figure 2 shows the distribution of food groups in term of storage in the overall countries and by regions. We observed a huge increase in storage for pasta, rice, or other grains, for water, and for flour. This increase was to a less extent for potatoes, legumes/pulses, bread, eggs, milk, and other dairy products. On the other hand, ready-made meals and fish (fresh, frozen and canned) were less stored during lockdown. Furthermore, lockdown did not show any impact on storage of fruits in any of its forms as well as vegetarian alternatives and salty snacks. When comparing the regions, we observed a high storage of flour (39%), pasta (38.4%), water (32.2%), and bread (30.9%) in the MENA countries. On the other hand, pasta (26.6%), water (26.2%), flour (23%), and milk (19%) were the more stored in the GCC countries.

#### Determinants of Food Insecurity in the Overall Population

Many factors affected the FCS of the people living in the EM countries studied. Before the pandemic, the percentage of people, living in the GCC countries, and having low FCS was 8% and increased to reach 9.3% during the pandemic. On the other hand, 10.6 and 13.6% of the MENA population studied had a low FCS before the pandemic and during the pandemic, respectively (Table 6). The determinants of food insecurity indicated by “low FCS” in the overall population, before and during the pandemic, is conditioned by many variables of which the region, gender, age categories, some cooking practices, cooking with leftovers and education. To explain, prior to the pandemic, the odds of low FCS in the MENA countries was 1.3 higher than in the GCC countries [OR:1.3; 95% CI (1.23- 1.56)]. Moreover, women were witnessing a FCS of 1.3 times higher than men [OR:1.3; 95% CI (1.19–1.59)]. Compared to adolescents (18 years), adults and elderly people were had a high FCS compared to younger people [OR:0.34; 95% CI (0.14-0.81)] and [OR:0.36; 95% CI (0.15–0.85)], respectively. Some cooking practices affected the FCS of the population studied. For instance, planning meals to include a variety of food [OR:1.4; 95% CI (1.21–1.64)], thinking about healthy choices when deciding the food choices [OR:1.2; 95% CI (1.06–1.47)], cooking meals at home using healthy ingredients [OR:1.3; 95% CI (1.10–1.57)], managing financially the healthy meal's content confidently [OR:1.4; 95% CI (1.17–1.68)], and cooking with leftovers [OR:1.17; 95% CI (1.02–1.33)], all together increased the FCS of around 1.4 odds compared to people who did not practiced these patterns. As for the education, it appears that people with high school diploma or bachelor's degree or master's degree had a 70% [OR:0.3; 95% CI (0.23–0.58)], 30% [OR:0.6; 95%CI (0.40–0.92)], and 35% [0.65; 95%CI (0.43–0.97)] higher FCS compared to those who attained lower levels of education. During the pandemic, the binary logistic regression analysis shows that people living in the MENA countries had a lower FCS of 1.5 times compared to those living in the GCC countries [OR:1.59; 95%CI (1.42–1.78)]. In addition, women were having a higher FCS of 1.11 times more [OR:1.11; CI (0.96–1.27)] compared with men. The age categories youth, adults and elderly had 60% [OR: 0.39; 95%CI (0.16–0.96)], 65% [OR: 0.39; 95%CI (0.16–0.96)], and 70% [OR: 0.39; 95%CI (0.16–0.96)], higher FCS respectively, compared with adolescent category. With regards to cooking practices, planning healthy meals [OR:1.34; 95%CI (1.15–1.57)], managing financially and confidently the preparation of healthy meals [OR:1.26; 95%CI (1.08–1.46)], cooking meals at home using healthy ingredients [OR:1.59; 95%CI (1.33–1.90)] and cooking with leftovers [OR:1.14; 95%CI (1.01–1.29)] all together had an impact on the FCS by increasing it in a range between 1.1 and 1.3 times. As for the educational level, people with higher education e.g., high school diploma and bachelor's degree had a higher FCS of 57% [OR:0.43; 95% CI (0.29–0.65)] and 35% [OR:0.65; 95% CI (0.45–0.93)] more compared to people with lower educational level. It was noticed that there was no impact of reading the nutritional panel, changing recipes to make them healthier, throwing away leftovers and employment on FCS, neither before nor during the lockdown (Table 6).

**Table 6:** Backwards Odds ratios (OR) according to food consumption score.

**Independent variable**	**Binary logistic regression**		
**Before lockdown**	**Odds ratio**	**OR confidence interval**	***p*** **-value**
Region: MENA vs. GULF	1.389	[1.230–1.569]	0
Gender: Female vs. Male	1.382	[1.196–1.598]	0
Age group: Youth vs. Adolescents	0.45	[0.184–1.101]	0.08
Age group: Adults vs. Adolescents	0.345	[0.146–0.813]	0.015
Age group: Elderly vs. Adolescents	0.367	[0.158–0.852]	0.02
Plan meals to include all food groups: positive vs. negative	1.413	[1.215–1.644]	0
Think about healthy choices when deciding what to eat: positive vs. negative	1.253	[1.066–1.474]	0.006
Feel confident about managing money to buy healthy food: positive vs. negative	0.873	[0.750–1.016]	0.078
Use the nutritional information panel: positive vs. negative	0.894	[0.758–1.055]	0.185
Use other parts of food label to make food choices: positive vs. negative	1.16	[0.983–1.367]	0.078
Cook meals at home using healthy ingredients: positive vs. negative	1.322	[1.109–1.575]	0.002
Feel confident about cooking a variety of healthy meals: positive vs. negative	1.41	[1.178–1.686]	0
Change recipes to make them healthier: positive vs. negative	1.061	[0.905–1.244]	0.463
Cook with leftover food: positive vs. negative	1.17	[1.029–1.331]	0.017
Throw away leftover food: positive vs. negative	0.938	[0.828–1.063]	0.318
Education level: High school diploma vs. under a high school diploma	0.374	[0.239–0.585]	0
Education level: Bachelor's degree vs. under a high school diploma	0.61	[0.402–0.926]	0.02
Education level: Master's degree vs. under a high school diploma	0.653	[0.438–0.974]	0.037
Education level: Doctorate vs. under a high school diploma	0.985	[0.634–1.531]	0.947
Employment status: I worked vs. I was a student	1.144	[0.927–1.411]	0.21
Employment status: I didn't work vs. I was a student	1.144	[0.961–1.363]	0.13
**During lockdown**	**Odds ratio**	**OR Confidence Interval**	***P*** **-value**
Region: MENA vs. GULF	1.594	[1.425–1.782]	0
Gender: Female vs. Male	1.111	[0.968–1.275]	0.133
Age group: Youth vs. Adolescents	0.399	[0.165–0.965]	0.042
Age group: Adults vs. Adolescents	0.355	[0.151–0.835]	0.018
Age group: Elderly vs. Adolescents	0.292	[0.126–0.677]	0.004
Plan meals to include all food groups: positive vs. negative	1.346	[1.150–1.576]	0
Think about healthy choices when deciding what to eat: positive vs. negative	1.09	[0.922–1.288]	0.313
Feel confident about managing money to buy healthy food: positive vs. negative	1.26	[1.085–1.463]	0.002
Use the nutritional information panel: positive vs. negative	0.903	[0.760–1.072]	0.243
Use other parts of food label to make food choices: positive vs. negative	1.045	[0.879–1.241]	0.618
Cook meals at home using healthy ingredients: positive vs. negative	1.593	[1.335–1.902]	0
Feel confident about cooking a variety of healthy meals: positive vs. negative	1.106	[0.922–1.327]	0.278
Change recipes to make them healthier: positive vs. negative	1.036	[0.884–1.213]	0.665
Cook with leftover food: positive vs. negative	1.147	[1.015–1.296]	0.028
Throw away leftover food: positive vs. negative	0.97	[0.862–1.091]	0.612
Education level: High school diploma vs. under a high school diploma	0.437	[0.294–0.651]	0
Education level: Bachelor's degree vs. under a high school diploma	0.652	[0.452–0.939]	0.022
Education level: Master's degree vs. under a high school diploma	0.742	[0.524–1.052]	0.094
Education level: Doctorate vs. under a high school diploma	1.07	[0.729–1.571]	0.73
Employment status: I worked vs. I was a student	0.898	[0.738–1.092]	0.28
Employment status: I didn't work vs. I was a student	1.081	[0.920–1.269]	0.344

## Discussion

The present study, the first of its kind in the region, aimed to assess the food consumption patterns, and the household's dietary diversity through the assessment of food consumption score and food-related patterns in 10 Eastern Mediterranean countries. At regional level, before and during the lockdown, the majority of food groups were consumed in a frequency of <3 times per week in the 10 Eastern Mediterranean countries: fruits (3 out of 5 persons), vegetables (1 over 2 persons), legumes and pulses (4 over 5 persons), nuts (3 out of 4 persons), unprocessed fish, poultry, and meats (3 out of 5 persons), milk and other dairy products (one over two persons), and added fats and oils (around one over 4 person). On the other hand, some food groups were consumed frequently (more than 4 times per week): each 13 over 50 persons were consuming processed meat/poultry/fish and vegetarian alternatives, each 9 over 20 persons consume sugary products, 3 out of 10 and 9 over 25 people were consuming wholewheat groups and white grains group, respectively. The confinement due to COVID-19 induced an increase in the consumption of legumes and pulses (3%, *p*-value = 0.00), nuts (1%, *p*-value = 0.009), unprocessed meats, poultry and fish (1–3%, *p*-value = 0.00), white grains group (2%, *p*-value = 0.00), whole wheat groups (3%, *p*-value = 0.00), milk (0.6%, *p*-value = 0.00), sugar (2%, *p*-value = 0.00), and added fats and oils (2%, *p*-value = 0.00). In contrast, a decrease of 4%, 6% and 0.9% was observed in the consumption of vegetables (*p*-value = 0.00), processed meats, poultry, and fish (*p*-value = 0.00) and other dairy products (*p*-value = 0.01), respectively. The consumption of fruits remained unchanged (*p*-value = 0.09). The FCS decreased of 3% in Jordan, 2.7% in Kuwait, 4.6% in Lebanon, 1.7% in Saudi Arabia and 3.2% in Palestine. It remained unchanged in UAE, Oman, Qatar, Bahrain, and Egypt. The most cooking practices (11 out of 13) showed a significant increase during the lockdown in the overall population and the proportions of food stocked have been changing since the start of COVID-19 and higher amounts of pasta, rice and other grains were stocked with an unchanged consumption rate of fruits and vegetables. Well, several challenges were observed throughout the epidemic such as absence of food, going to several places to find it, inability to afford some types, worries on food safety, and finding the best-price shops for buying some foods. Consumers tend to limit food types they cannot afford such as meat and fish and start consuming higher quantities of starchy food due to their wide availability and cheap prices (7). The mental status and anxiety related to food availability can push food insecure consumers toward more consumption of fruits, savory snacks, sweets, and candies which in turn can cause weight gain, as appeared in many other studies (9–11). Furthermore, COVID-19 pandemic was acquainted by a need for mitigation measures to compensate for household economic crisis. Consumers were forced to change their food consumption patterns involuntarily, rely on savings, sell household durable assets and livestock, buy foods with high shelf-life, eat less, buy cheaper food, and accept food from friend as a mean of mitigation measures (7). In many countries, physical distancing requirements and the international restrictions on travel along with the fear of disease have led to quarantining millions of people and affecting the global economy, social lives, tourism, and the hospitality industries that imposed disruption of supply chains for fresh produce, perishable and nutritious foods, such as fruits and vegetables, meat, milk and other dairy products that often requires many people to work in presence to cultivate, harvest and process. Moreover, it is affecting the production and transportation of manufactured food products. The pandemic also closed the informal markets exacerbating the inaccessibility of nutritious foods as well as livelihoods of vulnerable people. In high- and upper-middle-income countries, the cheap, highly processed, packaged foods with longer shelf life bombarded with high amounts of trans fats, fats, sugars, and salt may be consumed in higher amounts lowering by this the diet quality (1). Concerns have been raised regarding the impact of the COVID-19 curfew on food availability, consumption, access, and dietary diversity as part of food security in the EMR. The FCS is a composite score that evaluate the dietary diversity and food frequency when assessing food security. The decrease in the FCS in the five countries: Jordan, Kuwait, Lebanon, Saudi Arabia, and Palestine may be related to several factors such as the critical political and economic situations affecting the region with a more important decrease in the MENA countries compared to the GCC region (7). Beyond the causes of food insecurity in the Arab region, clashes, tensions and civil insecurity have remained the foremost critical drivers of food insecurity nowadays due to numerous factors such as razing farm land, killing live stocks, bombs, blocking access to markets, refugee migration in addition to ongoing other conflicts (12). In this study, other factors were influencing the FCS in different directions. Specifically, as planning meals and giving many concerns to variety foods, education level and money management to buy healthy food during the lockdown varied, FCS increased too. Whereas, as region, gender and managing money to buy healthy food before the lockdown changes, FCS decreased. All these factors may be directly related to meal organization abilities, knowledge, economic status, person responsible for preparing food and the cultural differences between countries in term of food consumption. To mitigate the effects of income losses and decrease in purchasing power, people started to adjust to these shortages through different mitigation strategies. Among these strategies, a significant change in most of cooking practices was observed such as cooking more with leftovers in addition to minimizing food wastes. Furthermore, COVID-19 had led people suffer a significant increase in barriers to cooking healthy meals due to the unavailability of money and less accessibility to food and cooking facilities. During the curfew, people searched more for inexpensive recipes that can be achieved with fewer ingredients throughout the cooking process. These can be explained by the financial struggle communities are having to preserve food leftovers rather than throwing them away and ensuring enough resources for healthy cooking with a high emphasis on the cultural role of females in cooking. Even when purchasing ingredients, people tend to search more for cheaper prices and less organic foods but order them online rather than going physically to the stores (13–17). These statements came hand by hand with our findings where the most cooking practices (11 out of 13) showed a significant increase during the lockdown. Besides, proportions of food stocked have been changing since the start of COVID-19 and higher amounts of pasta, rice and other grains were stocked with an unchanged consumption rate of fruits and vegetables, due to their longer shelf life and easier storage conditions. This may be related to the need for these food groups and their cheap prices as considered as staple foods especially in the EMR.

### Comparison With Other International Countries

When comparing our results with other countries' findings, we observed that the pandemic caused some modifications in food-related activities in Russia, of which a decrease of meat consumption and sweet products was observed in 1,047 adults along with an adoption of healthier consumption patterns (18). In addition, in an American survey (*n* = 484 adult participants), food insecure respondents were reducing their fruits and vegetables consumption since the start of the pandemic, and they perceived cost as barrier to eating these foods (19). The findings of the American study, along with our findings, came hand in hand with 2 Italian surveys in which 18% of the respondents reported consuming fewer fresh fruits while in the other study 8.7% of the respondents reported consuming fewer fresh fruits and vegetables (20, 21). Similarly, in Italy, 2,768 adults showed an improvement in the diet quality along with an increase in the consumption of fruit (24.4%), vegetables (28.5%), legumes (22.1%), nuts (12%), and fish or shellfish (14%). However, it was observed that the excessive consumption of sweets or pastries (36.9%) increased during the lockdown (22). According to Enriquez-Martinez et al., the survey conducted in Argentina, Brazil, Mexico, Peru, and Spain on 6,325 adults shows that most participants (61.6%), mainly those from Spain didn't show any improving or worsening in their food pattern. Argentina and Brazil showed a high improvement toward a healthier food consumption patterns. Fewer changes in food consumption patterns were observed among Peruvians and Mexicans (OR: 0.51; 95% CI: 0.4–0.6 and OR: 0.69; 95% CI: 0.4–0.8, respectively), when compared to Argentinians (23). A Polish survey enrolled in Poland (*n* = 2,381 adults) shows that 30% of respondents improved their food patterns through increased intake of vegetables, milk and milk products. In addition, 75%, 50% and 20% of the respondents reduced their intake of fast food and commercial pastry, of confectionary and salty snacks, and of sugar-sweetened beverages and alcohol, respectively (24). In France, the COVID-19 pandemic strongly affected the nutritional quality of the respondents' diet (*n* = 938) during the first lockdown. Moreover, an increase in the consumption of fruits, vegetables, pulses, fish and seafood was observed along with a sharp increase in processed meat, sweet-tasting beverages and alcoholic beverages consumption that negatively affected the quality of diet (25). In addition, a French web-based survey that encompassed 37,252 adults showed a reduction in the intake of fresh products of which 17% of participants decreased their consumption of fresh fruits, 18% for fresh vegetables, 22% for fresh red meats, and 31% for fresh fish. On the other hand, 22% of people increased their consumption of sweets and chocolate, cookies, and cakes (20%) and cheese (18%) along with a decreased consumption of sandwiches, pizzas, or savory pies (17%) (26). The Lithuanian COVIDiet Study (*n* = 2,447 participants) showed an increment in the frequency of consumption of sweets, biscuits, and cakes associated with a reduction in fruits and vegetables intake and increased consumption of frozen and canned foods. In addition, more frequent cooking and eating out of control were reported by the study participants. On the other hand, processed meats and carbonated or sugary drinks were less consumed (27). The findings from a survey conducted by three European countries on a total of 1,071 adults: from Poland (*n* = 407), Austria (*n* = 353) and the United Kingdom (*n* = 311) showed an increased frequency in purchasing frozen goods and food with long shelf life and in the daily intake of dairy products, grains, fats, vegetables, and sweets (28). In Belgium, an online questionnaire interviewed 8,640 adults where 10.4% of Belgians were showing food shortages, 5 % had limited monetary access to food and 10·3 % couldn't afford eating a healthy diet during lockdown. This status of food insecurity was associated with a change in most dietary behaviors (29). The people living in Denmark, Germany and Slovenia witnessed, during the pandemic, a reduction in their consumption of fresh food. This change was due to the decrease in the frequency of shopping in all the three countries and women were more likely to increase their consumption of fresh fruits compared with men (30). Three studies in Brazil were conducted between 2020 and 2021 in which more than 50,000 adults were interviewed through web-based questionnaires. The first study showed an increase in the consumption of high energy density foods (potato fries, chocolate, and ice cream) and ultra-processed foods among Brazilian adults (31). In the second study, an increase in the consumption of vegetables, fruits, and legumes along with a stability in the consumption of ultra-processed foods was observed (32). The third study revealed a decrease in the consumption of fruits and vegetables along with an increase in the consumption of candies and fast-food (33). In Chile (*n* = 700 adults), negative eating habits were dominating, such as low consumption of legumes and water and high consumption of junk food (e.g., food with low food quality, low contribution of micronutrients and with a high contribution of sugar, saturated fat, and sodium) and fried foods (34). In China (*n* = 2,702 adults), no changes were observed in the habitual diet, while 38.2% of participants reported an increase in their snack intake, during the lockdown. These results were interpreted that basic food supplies were guaranteed in China since the start of the lockdown. These findings were consistent with another cross-sectional study among adults in Netherlands where 83% of participants reported no change in their eating patterns during the COVID-19 lockdown (35). A scoping review was designed to assess the literature on the impact of lockdown on dietary changes in various population groups (US, Asia including Palestine, India, and China, Europe including Italy, France, Spain, Poland, and the UK, Australia, and Zimbabwe) in which in a total of ten studies an increase in the number of snacks consumed was observed, while in six studies, participants increased the quantity and the frequency of meal intakes during quarantine. Eleven studies reported improvement in dietary habits, an increase in fresh produce and home cooking along with a decrease in the intake of alcohol and comfort food. Additional nine studies found a decrease in fresh produce, with a further six showing an increase in comfort foods intake including sweets, fried food, snack foods, and processed foods (36).

### Comparison With Other Regional Countries

At regional level, in Jordan, among a total of 3129 Jordanians, 23.1% were severely affected by food insecurity, 36.1% were moderate food insecure and 40.7% were food secure. Carbohydrates and the meat group were significantly related to food insecurity (*p*-value was <0.001 for both groups) where food insecure people were consuming fewer meats and carbohydrates compared to food secure people (9). In addition, the impact of COVID-19 pandemic on food purchasing and dietary behaviors was studied in three Kuwaiti surveys on 1,935 respondents in Kuwait. In the first study, the consumption of vegetables, fruits, and carbohydrates increased. It was associated with a decreased consumption of fish and sugary drinks (37). Otherwise, in the second study, no significant differences were found before and during the lockdown in terms of the weekly frequency of food groups consumption, except in the case of fish and seafood (14). One over two participants in the third study indicated that their food consumption patterns remained unchanged (44%). In addition, 50.3% reported that they ate more fruits and vegetables, legumes, and pulses (41.5%), and fewer fast food (2.3%) (15). In Lebanon, 44.7% and 35% of study participants weren't eating fruits and vegetables daily, 28% reported consuming sweets or desserts once per day, 30.9% consumed salty snacks (nuts, crackers, chips) each day and 24.7% of people consumed sweetened drinks at least once per day (16). Moreover, according to Hoteit et al., 9 in every 16 households ate <2 meals per day and more than 70% of them skipped their meals to spare food. Even though half the Lebanese population studied had a low food consumption score (7). Another study conducted in three countries of which Lebanon was included, showed that 33% and 31% of the respondents stated that they shopped for food once a week and two to three times a week during the COVID-19 pandemic, respectively. In comparison, 25% purchased food less than once a week. Obtaining food and groceries by supermarkets or food shops' delivery services was not common in the three countries (10%) (Lebanon, Tunisia, and Jordan) and was significantly the least reported among the Tunisians (17). All in all, it appears that households with dietary restrictions were more likely to experience both pre-pandemic and pandemic-related incident or worsening food insecurity than households without restrictions (38).

## Limitations

The present study aimed at investigating one of the accurate proxy indicators of food security, the FCS. Some limitations should be considered when evaluating the results of this study. It included retrospective data that was based on the respondents' memory to recall the food groups consumption and patterns before the lockdown, which may affect the presented eating habits. The questionnaire was quite long and was more often completed by people with higher education who had access to good quality of internet.

## Conclusion

Due to the social isolation implemented during the COVID-19 pandemic, some changes in the food consumption patterns occurred in the study population. The changes were remarkable in the frequency of consumption of food products such as vegetables and other dairy products e.g., cheese. No increase in the consumption of fruits was observed. Nevertheless, an increase in the consumption of legumes and pulses, nuts, meats, poultry, fish, white bread, whole wheat bread, and milk occurred. In the short term, with some exceptions, the results obtained may suggest that nutrition patterns did not change much during lockdown, nor does it affect much the frequency of consumption of healthy products in the diet. Nerveless, the most cooking practices showed a significant increase during the lockdown in the overall population and the proportions of food stocked have been changing since the start of the pandemic where higher amounts of pasta, rice, and other grains were stocked with an unchanged storage rate of fruits and vegetables. To conclude, the 2020 COVID-19 crisis revealed how unprepared the region was to respond appropriately to the pandemic. It showed in particular how supply chains vary in complexity and vulnerability to disruption. Their capacity to respond effectively will depend on the resilience of the distribution chains, and the readiness to improve. As such, a definitive capacity to ricochet back and recuperate from a shock doesn't rely exclusively upon the force/seriousness of the underlying shock, however on the effect of that shock's joined with the reactions that entertainers (independently, or as networks or society) set up to alleviate or check the underlying impact of that shock. Additionally, building strength and resilience in food systems and frameworks is tied in with building capacities. For the greater part of the entertainers in the EMR's local food system, creating capacities that are more in accordance with the qualities and casualness of their current circumstance will require more planned research. Better admittance to data, more grounded participation, more incorporation, and more elevated levels of earning and self-adequacy for those entertainers will make the local nations personally reliant upon one another which helps adopting a regional food system resilience framework that assists in better understanding the intricacy of the circumstance and the potential expanding influences which might go through the whole food system once one part is affected later.

## Data Availability Statement

The original contributions presented in the study are included in the article/Supplementary Material, further inquiries can be directed to the corresponding author/s.

## Ethics Statement

The study was conducted according to the guidelines of the Declaration of Helsinki, and approved by the Ethical Committee for the Social Sciences and Humanities of the University of Antwerp (file number 20_46) as well as in all other concerned countries. The patients/participants provided their written informed consent to participate in this study.

## Corona Cooking Survey Regional Study Group

Belgium: Charlotte De Backer, Kathleen Van Royen, Lauranna Teunissen, Isabelle Cuykx, Paulien Decorte, Gaëlle Ouvrein, Karolien Poels, Heidi Vandebosch, Katrien Maldoy (University of Antwerp); Sara Pabian (Tilburg University), Christophe Matthys, Tim Smits, Jules Vrinten (KULeuven); Ann DeSmet (University of Antwerp, Université Libre de Bruxelles); Nelleke Teughels, Maggie Geuens, Iris Vermeir, Viktor Proesmans, Liselot Hudders (Ghent University); Bahrain: Tariq Abdulkarim Alalwan (University of Bahrain); Jordan: Nahla Al-Bayyari (Al-Balqa Applied University), Mohammed O. Ibrahim (Mu'tah University), Fadwa Hammouh (American University of Madaba); Kuwait: Basma Dashti (Kuwait Institute for Scientific Research), Dhuha Alkharaif (Public Authority for Applied Education & Training), Amani Alshatti (Public Authority for Applied Education & Training), Maryam Al Mazedi (Public Authority for Applied Education & Training); Lebanon: Elissa Naim (Lebanese University), Carla Ibrahim (Lebanese University & Holy Spirit University of Kaslik); Palestine: Motasem Hamdan, Diala Abu Al Halawa, Hazem Agha (Al Quds University); Qatar: Manal Othman (Hamad Medical Corporation), Jaafar Pakari, Allam Abu Farha, Rasha Abu-El-Ruz (Qatar University; QU-Health); Saudi Arabia: Jamila Arrish [National Nutrition Committee (NNC) at Saudi Food and Drug Authority (Saudi FDA)]; United Arab Emirates: Zainab Taha (Zayed University), Ayesha Aldhaheri (United Arab Emirates University).

## Author Contributions

CB, LT, IC, PD, SP, and KR: conceptualization, software, validation, supervision, project administration, and funding acquisition. CB, LT, IC, PD, SP, KR, MH, HM, AJ-J, RM, MA, KB, RT, HA, LC, RQ, RA, IK, SD, SA, MA-M, HB, MW: methodology. MH and HM: formal analysis, data curation, and writing—original draft preparation. MH: investigation. CB, LT, IC, PD, SP, KR, and AJ-J: resources. All authors and the research group contributed to the article and approved the submitted version.

## Funding

This research was funded by the Research Foundation Flanders (G047518N) and Flanders Innovation and Entrepreneurship (HBC.2018.0397). These funding sources had no role in the design of the study, the analysis and interpretation of the data or the writing of, nor the decision to publish the manuscript.

## Author Disclaimer

The views expressed in this paper are those of the author(s) and do not necessarily reflect those of the WHO and SFDA or its stakeholders. Guaranteeing the accuracy and the validity of the data is a sole responsibility of the research team.

## Conflict of Interest

The authors declare that the research was conducted in the absence of any commercial or financial relationships that could be construed as a potential conflict of interest.

## Publisher's Note

All claims expressed in this article are solely those of the authors and do not necessarily represent those of their affiliated organizations, or those of the publisher, the editors and the reviewers. Any product that may be evaluated in this article, or claim that may be made by its manufacturer, is not guaranteed or endorsed by the publisher.
