# Supplementary material for: Dietary Diversity in the Eastern Mediterranean Region Before and During the COVID-19 Pandemic: Disparities, Challenges, and Mitigation Measures
Source: Front Nutr. 2022 Feb 15;9:813154. doi: 10.3389/fnut.2022.813154 (PMC8893198; doi:10.3389/fnut.2022.813154)
Supplement: Supplementary file 1 [file Table_1.DOCX]

Supplementary Material

|  | Overall Before N  (%) | | Overall During N  (%) | | Variation (%) | *p*-value | GCC before N  (%) | | GCC during N  (%) | | MENA before N  (%) | | MENA during N  (%) | | *p*-value | |
| --- | --- | --- | --- | --- | --- | --- | --- | --- | --- | --- | --- | --- | --- | --- | --- | --- |
|  | No | Yes | No | Yes |  |  | No | Yes | No | Yes | No | Yes | No | Yes | Before | During |
| Plan meals to include all food groups (A varied diet) | 4941 (36.5) | 8586 (63.5) | 4299 (31.8) | 9228 (68.2) | 4.7 | 0 | 2639  (37.8) | 4338  (62.2) | 2178 (31.2) | 4799 (68.8) | 2302 (35.1) | 4248 (64.9) | 2121 (32.4) | 4429 (67.6) | 0.001 | 0.146 |
| Think about healthy choices when deciding what to eat | 4225 (31.2) | 9302 (68.8) | 3875 (28.6) | 9652 (71.4) | 2.6 | 0 | 2111  (30.3) | 4866  (69.7) | 1907 (27.3) | 5070 (72.7) | 2114 (32.3) | 4436 (67.7) | 1968 (30.0) | 4582 (70.0) | 0.011 | 0 |
| Feel confident about managing money to buy healthy food | 4269 (31.6) | 9258 (68.4) | 3977 (29.4) | 9550 (70.6) | 2.2 | 0 | 2260  (32.4) | 4717  (67.6) | 2021 (29.0) | 4956 (71.0) | 2009 (30.7) | 4541 (69.3) | 1956 (29.9) | 4594 (70.1) | 0.031 | 0.253 |
| Use the nutritional information panel (nutritional breakdown of the products) to make food choices | 6606 (48.8) | 6921 (51.2) | 5977 (44.2) | 7550 (55.8) | 4.6 | 0 | 3303  (47.3) | 3674  (52.7) | 3029 (43.4) | 3948 (56.6) | 3303 (50.4) | 3247 (49.6) | 2948 (45.0) | 3602 (55.0) | 0 | 0.062 |
| Use other parts of food label to make food choices | 6358 (47) | 7169 (53) | 5691 (42.1) | 7836 (57.9) | 4.9 | 0 | 3144  (45.1) | 3833  (54.9) | 2891 (41.4) | 4086 (58.6) | 3214 (49.1) | 3336 (50.9) | 2800 (42.7) | 3750 (57.3) | 0 | 0.122 |
| Cook meals at home using healthy ingredients | 3062 (22.6) | 10465 (77.4) | 3013 (22.3) | 10514 (77.7) | 0.3 | 0.3 | 1648  (23.6) | 5329  (76.4) | 1568 (22.5) | 5409 (77.5) | 1414 (21.6) | 5136 (78.4) | 1445 (22.1) | 5105 (77.9) | 0.005 | 0.564 |
| Feel confident about cooking a variety of healthy meals | 2987 (22.1) | 10540 (77.9) | 2965 (21.9) | 10562 (78.1) | 0.2 | 0.6 | 1632  (23.4) | 5345  (76.6) | 1541 (22.1) | 5436 (77.9) | 1355 (20.7) | 5195 (79.3) | 1424 (21.7) | 5126 (78.3) | 0 | 0.626 |
| Change recipes to make them healthier | 4241 (31.4) | 9286 (68.6) | 3688 (27.3) | 9839 (72.7) | 4.1 | 0 | 2172  (31.1) | 4805  (68.9) | 1893 (27.1) | 5084 (72.9) | 2069 (31.6) | 4481 (68.4) | 1795 (27.4) | 4755 (72.6) | 0.567 | 0.722 |
| Cook with leftover food | 5355 (39.6) | 8172 (60.4) | 4643 (34.3) | 8884 (65.7) | 5.3 | 0 | 2810  (40.3) | 4167  (59.7) | 2493 (35.7) | 4484 (64.3) | 2545 (38.9) | 4005 (61.1) | 2150 (32.8) | 4400 (67.2) | 0.091 | 0 |
| Throw away (leftover) food | 8949 (66.2) | 4578 (33.8) | 9126 (67.5) | 4401 (32.5) | -1.3 | 0 | 4665  (66.9) | 2312  (33.1) | 4748 (68.1) | 2229 (31.9) | 4284 (65.4) | 2266 (34.6) | 4378 (66.8) | 2172 (33.2) | 0.073 | 0.133 |
| Money to buy healthy diet (I didn't have the funds for the foods/ingredients I needed or wanted) | 4224 (68.7) | 1928 (31.3) | 4049 (65.8) | 2103 (34.2) | 2.9 | 0 | 2500  (73.8) | 886  (26.2) | 2486 (73.4) | 900 (26.6) | 1724 (62.3) | 1042 (37.7) | 1563 (56.5) | 1203 (43.5) | 0 | 0 |
| Access to food (I didn't have access to foods/ingredients I needed or wanted) | 4258 (69.2) | 1894 (30.8) | 3764 (61.2) | 2388 (38.8) | 8 | 0 | 2469  (72.9) | 917  (27.1) | 2253 (66.5) | 1133 (33.5) | 1789 (64.7) | 977 (35.3) | 1511 (54.6) | 1255 (45.4) | 0 | 0 |
| Access to cooking facilities (I didn't have (access to) the facilities needed to cook or bake: stove, oven, kitchen equipment) | 4669 (75.9) | 1483 (24.1) | 4461 (72.5) | 1691 (27.5) | 3.4 | 0 | 2656  (78.4) | 730  (21.6) | 2605 (76.9) | 781 (23.1) | 2013 (72.8) | 753 (27.2) | 1856 (67.1) | 910 (32.9) | 0 | 0 |

Table S1. Mitigation measures related to cooking attitudes, practices, and barriers in the overall population and by regions.

**Table S2.** Mitigation measures related to recipes selections, overall and by regions.

| Selection  of recipes | Overall Before  N  (%) | | | Overall During  N  (%) | | | *p*-value | Overall Variation | | GCC before  N  (%) | | | GCC during  N  (%) | | | MENA before  N  (%) | | | MENA during  N  (%) | | | *p*-value | |
| --- | --- | --- | --- | --- | --- | --- | --- | --- | --- | --- | --- | --- | --- | --- | --- | --- | --- | --- | --- | --- | --- | --- | --- |
|  | Disagree. | Neutral | Agree | Disagree | Neutral | Agree |  | Disagree | Agree | Disagree | Neural | Agree | Disagree | Neutral | Agree | Disagree | Neutral | Agree | Disagree | Neutral | Agree | Before | During |
| Achievable with few ingredients | 1276 (20.7) | 1312 (21.3) | 3564 (58) | 735 (11.9) | 1019 (16.6) | 4398 (71.5) | 0 | -8.8 | 13.5 | 666 (19.7) | 742 (21.9) | 1978  (58.4) | 386  (11.4) | 570 (16.8) | 2430 (71.8) | 610 (22.1) | 570 (20.6) | 1586 (57.3) | 349 (12.6) | 449 (16.2) | 1968 (71.1) | 0 | 0 |
| Achievable with the ingredients at home | 724 (11.8) | 772 (12.5) | 4656 (75.7) | 561  (9.1) | 706 (11.5) | 4885 (79.4) | 0 | -2.7 | 3.7 | 377 (11.1) | 439  (13) | 2570  (75.9) | 295  (8.7) | 379 (11.2) | 2712 (80.1) | 347 (12.5) | 333  (12) | 2086 (75.4) | 266  (9.6) | 327 (11.8) | 2173 (78.6) | 0 | 0 |
| Achievable with ingredients that can be easily found at the store | 643 (10.5) | 686 (11.1) | 4823 (78.4) | 573  (9.3) | 700 (11.4) | 4879 (79.3) | 0.007 | -1.2 | 0.9 | 348 (10.3) | 392 (11.6) | 2646  (78.1) | 305  (9) | 379 (11.2) | 2702 (79.8) | 295 (10.7) | 294 (10.6) | 2177 (78.7) | 268  (9.7) | 321 (11.6) | 2177 (78.7) | 0 | 0 |
| Inexpensive to prepare | 962 (15.6) | 1323 (21.5) | 3867 (62.9) | 802 (13.1) | 1165 (18.9) | 4185 (68) | 0 | -2.5 | 5.1 | 482 (14.2) | 759 (22.4) | 2145  (63.3) | 425  (12.6) | 664 (19.6) | 2297 (67.8) | 480 (17.4) | 564 (20.4) | 1722 (62.3) | 377  (13.6) | 501 (18.1) | 1888 (68.3) | 0 | 0 |
| Healthy | 729 (11.9) | 1067 (17.3) | 4356 (70.8) | 656 (10.7) | 1017 (16.5) | 4479 (72.8) | 0 | -1.2 | 2 | 409 (12.1) | 628 (18.5) | 2349  (69.4) | 382  (11.3) | 594 (17.5) | 2410 (71.2) | 320 (11.6) | 439 (15.9) | 2007 (72.6) | 274  (9.9) | 423 (15.3) | 2069 (74.8) | 0 | 0 |
| Good (cheapest) prices | 1051 (19.5) | 907 (16.8) | 3433 (63.7) | 1023 (19) | 876 (16.2) | 3492 (64.8) | 0.101 | -0.5 | 1.1 | 605 (20.4) | 538 (18.1) | 1828  (61.5) | 618  (20.8) | 536 (18.0) | 1817 (61.2) | 446 (18.4) | 369 (15.2) | 1605 (66.3) | 405  (16.7) | 340 (14.0) | 1675 (69.2) | 0 | 0.001 |

**Table S3.** Dietary shopping practices before and during the pandemic, overall and by regions.

|  |  |  | Before  N  (%) | | During  N  (%) | | Variation  (%) | *p*-value | GCC before  N  (%) | | GCC during  N  (%) | | MENA before  N  (%) | | MENA during  N  (%) | | *p*-value | |
| --- | --- | --- | --- | --- | --- | --- | --- | --- | --- | --- | --- | --- | --- | --- | --- | --- | --- | --- |
|  |  |  | No | Yes | No | Yes |  |  | No | Yes | No | Yes | No | Yes | No | Yes | B | D |
| Organization of groceries shopping | Reach physically the supermarket, shop, market, farmer, vendor to select and buy food. | | 677  (12.6) | 4714 (87.4) | 1368  (25.4) | 4023 (74.6) | -12.8 | 0 | 364  (12.3) | 2607 (87.7) | 875  (29.5) | 2096 (70.5) | 313  (12.9) | 2107 (87.1) | 493  (20.4) | 1927 (79.6) | 0.452 | 0 |
|  | Online food order and pick it up at a seller's point. | | 3771 (69.9) | 1620 (30.1) | 3706  (68.7) | 1685 (31.3) | 1.2 | 0.058 | 1974  (66.4) | 997  (33.6) | 1980 (66.6) | 991  (33.4) | 1797  (74.3) | 623  (25.7) | 1726 (71.3) | 694  (28.7) | 0 | 0 |
|  | Online food order with delivery at home. | | 3041 (56.4) | 2350 (43.6) | 2801  (52) | 2590  (48) | 4.4 | 0 | 1448 (48.7) | 1523 (51.3) | 1334 (44.9) | 1637  (55.1) | 1593 (65.8) | 827  (34.2) | 1467 (60.6) | 953  (39.4) | 0 | 0 |
| Places of groceries shopping | Supermarket | | 543  (10.1) | 4848 (89.9) | 1021  (18.9) | 4370 (81.1) | -8.8 | 0 | 289  (9.7) | 2682 (90.3) | 612  (20.6) | 2359 (79.4) | 254  (10.5) | 2166 (89.5) | 409  (16.9) | 2011 (83.1) | 0.351 | 0.001 |
|  | Corner store/convenience store | | 1455  (27) | 3936  (73) | 1782  (33.1) | 3609 (66.9) | -6.1 | 0 | 986  (33.2) | 1985 (66.8) | 1218  (41) | 1753  (59) | 469  (19.4) | 1951 (80.6) | 564  (23.3) | 1856 (76.7) | 0 | 0 |
|  | Organic/ Fairtrade food shop | | 2787 (51.7) | 2604 (48.3) | 3512  (65.1) | 1879 (34.9) | -13.4 | 0 | 1570 (52.8) | 1401 (47.2) | 2040  (68.7) | 931  (31.3) | 1217 (50.3) | 1203 (49.7) | 1472  (60.8) | 948  (39.2) | 0.062 | 0 |
|  | Straight from the farmer / producer | | 3513 (65.2) | 1878 (34.8) | 3672  (68.1) | 1719 (31.9) | -2.9 | 0 | 1911 (64.3) | 1060 (35.7) | 2115  (71.2) | 856  (28.8) | 1602 (66.2) | 818  (33.8) | 1557 (64.3) | 863  (35.7) | 0.150 | 0 |
|  | Specialty stores: bakery, butcher, delicacy shop | | 1352 (25.1) | 4039 (74.9) | 2120  (39.3) | 3271 (60.7) | -14.2 | 0 | 889  (29.9) | 2082  (70.1) | 1444 (48.6) | 1527  (51.4) | 463  (19.1) | 1957 (80.9) | 676  (27.9) | 1744 (72.1) | 0 | 0 |
|  | Via meal kits/ meal boxes | | 3846 (71.3) | 1545 (28.7) | 4018  (74.5) | 1373 (25.5) | -3.2 | 0 | 2166  (72.9) | 805  (27.1) | 2295 (77.2) | 676  (22.8) | 1680 (69.4) | 740  (30.6) | 1723  (71.2) | 697  (28.8) | 0.005 | 0 |
